# Hygienisation and Nutrient Conservation of Sewage Sludge or Cattle Manure by Lactic Acid Fermentation

**DOI:** 10.1371/journal.pone.0118230

**Published:** 2015-03-18

**Authors:** Hendrik A. Scheinemann, Katja Dittmar, Frank S. Stöckel, Hermann Müller, Monika E. Krüger

**Affiliations:** 1 Institute of Bacteriology and Mycology, University of Leipzig, Faculty of Veterinary medicine, An den Tierkliniken 29, 04103 Leipzig, Germany; 2 Institute of Parasitology, University of Leipzig, Faculty of Veterinary medicine, An den Tierkliniken 35, 04103 Leipzig, Germany; 3 Institute of Virology, University of Leipzig, Faculty of Veterinary medicine, An den Tierkliniken 29, 04103 Leipzig, Germany; 4 Gesellschaft zur Förderung von Medizin-, Bio- und Umwelttechnologien e. V. Erich-Neuß-Weg 5, 06120 Halle (Saale), Germany; Purdue University, UNITED STATES

## Abstract

Manure from animal farms and sewage sludge contain pathogens and opportunistic organisms in various concentrations depending on the health of the herds and human sources. Other than for the presence of pathogens, these waste substances are excellent nutrient sources and constitute a preferred organic fertilizer. However, because of the pathogens, the risks of infection of animals or humans increase with the indiscriminate use of manure, especially liquid manure or sludge, for agriculture. This potential problem can increase with the global connectedness of animal herds fed imported feed grown on fields fertilized with local manures. This paper describes a simple, easy-to-use, low-tech hygienization method which conserves nutrients and does not require large investments in infrastructure. The proposed method uses the microbiotic shift during mesophilic fermentation of cow manure or sewage sludge during which gram-negative bacteria, enterococci and yeasts were inactivated below the detection limit of 3 log_10_ cfu/g while lactobacilli increased up to a thousand fold. Pathogens like *Salmonella*, *Listeria monocytogenes*, *Staphylococcus aureus*, *E*. *coli* EHEC O:157 and vegetative *Clostridium perfringens* were inactivated within 3 days of fermentation. In addition, ECBO-viruses and eggs of *Ascaris suum* were inactivated within 7 and 56 days, respectively. Compared to the mass lost through composting (15–57%), the loss of mass during fermentation (< 2.45%) is very low and provides strong economic and ecological benefits for this process. This method might be an acceptable hygienization method for developed as well as undeveloped countries, and could play a key role in public and animal health while safely closing the nutrient cycle by reducing the necessity of using energy-inefficient inorganic fertilizer for crop production.

## Introduction

**Fig 1:**
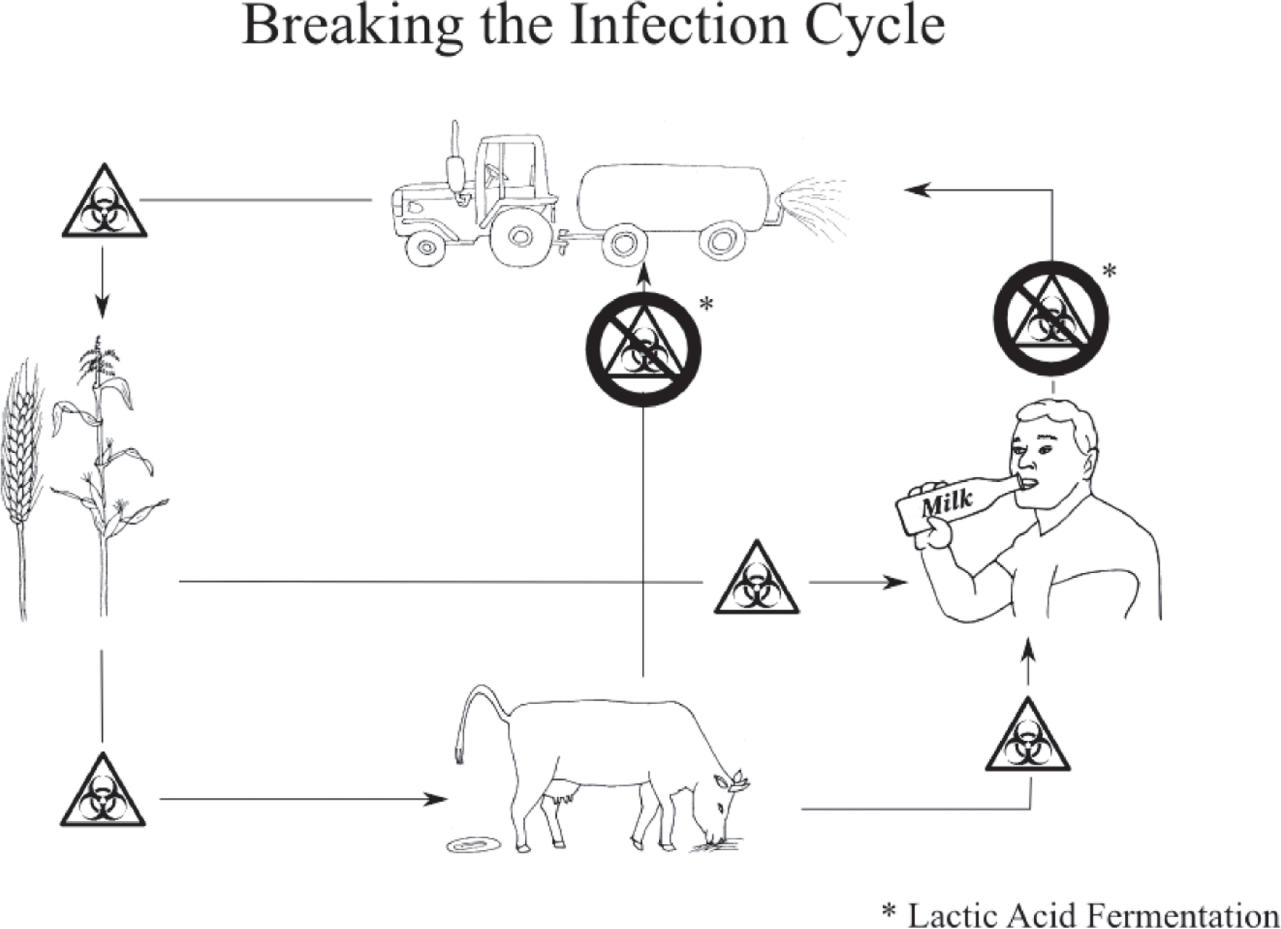
Graphical abstract.

Pathogens and various facultative organisms can be found in high concentrations in the faeces of animals and humans. Used as fertilizers, these materials can contaminate soil [1,2] as well as field crops [3–7]. Contaminated crops can, in turn, infect consumers [8] to complete the cycle of infection [9] as shown in Fig. 1. Interruption of these cycles is one objective of our research in order to facilitate better usage of these organic nutrient resources. Techniques commonly used to decrease the infectious potential of manures vary in effectiveness. Bagge et al. describe findings of low concentrations of enterococci in after digestion in biogas plants and a resettlement of enterococci and coliforms during storage of digested material [10]. Pourcher et al. describe a reduction of coliforms and enterococci during composting within months, but they still maintain a medium residual concentration [11]. Besides composting, with its huge mass loss [12] and greenhouse gas production [13] during the self heating phase, or pasteurization before methanogene or hydrogen digestion, no other methods are widely used in Germany.

Lactic acid fermentation has been used since the Stone and Bronze Age [14,15] to make dough and food, as well as to conserve fodder [16]. The developing *Lactobacillaceae* flora prevents oxidation and the development of pathogens within the fermented goods [17] by producing volatile fatty acids (VFA). The VFA can be bactericidal [18–21], depending on pH [22]. The lactic acid faeces fermentation used in this study is modelled after the suppositional conception of the native Amazonian [23] inhabitants who produced an explicitly good soil, the so-called *terra preta do indio*, thousands of years ago [24]. Moreover, they lived in large settlements [25] that imply the obvious need for an adequate hygienisation and nutrient recycling-system which could work in the humid tropics. Even today, some German farmers remember an old country saying of their grandparents which strongly suggests that earlier generations profited from lactic fermentation in their dunghills: “Schicht in gut, halt ihn feucht, tritt ihn fest, das ist für den Mist das Allerbest.” It means: “Layer it well, keep it wet, tread it well; that is best for dunghills.” Hygienisation via lactic acid fermentation has not been investigated for faeces previously.

Therefore a stand-alone method for the inactivation of pathogens was tested, independent of the later direct use as a fertilizer or as biogas-plant substrate.

To test the hygienisation potential of this biological method, bacterial, viral and parasitic pathogens were fermented in animal manure or sewage sludge for various times. Furthermore VFA concentrations, pH and mass loss during fermentation were measured.

## Materials and Methods

**Fig 2:**
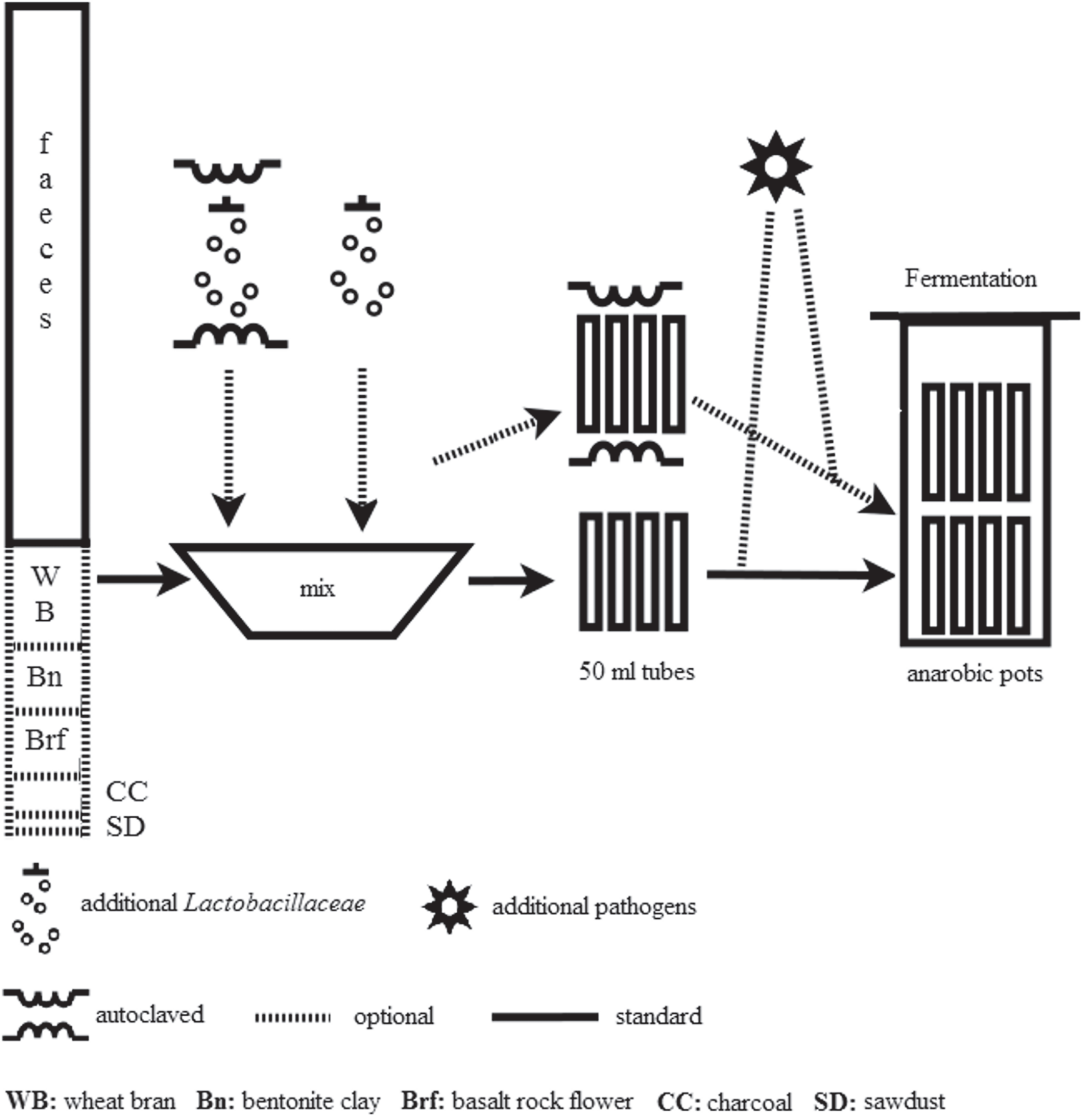
Experimental setup.

As shown in Fig. 2 sketchily, different faecal materials were fermented in 50 ml tubes (TPP, Switzerland, Trasadingen) and 27 g of each waste material plus 3 ml of pathogen or control suspension were added to each tube. It was incubated at 37°C under anaerobic conditions in Anaerocult pots and kits (Merck Germany, Darmstadt) for 3 to 56 days. After incubation, we analyzed the bacterial flora, virus titre and rate of embryonation of *Ascaris* eggs.

### Up- and Down- scaling

For safety reasons in working with pathogens an experimental size of 27 g was chosen. Moreover satellite experiments without pathogens using 1 litre (37°C) and 60 litre (27°C) containers were performed.

### Treatments

Archaeological findings show, that the terra preta made by native Amazonian culture consists basicaly of organic wastes and residue of fire, namely ash, fish, game and human bones, shells, urine and feces. Furthermore, pottery and stone implements covered the ground of the terra preta sites [26] so it seems obvious that they may have transported their organic waste within crocks to those sites. A consequence of storing organic wastes and residues of fire within crocks could have been a self establishing lactic acid fermentation process.

The design of the faecal material should resemble a typical waste mixture of a veterinary hospital. The treatments consisted mainly of manure from a dairy farm (Versuchsgut Oberholz, Großpösna, Germany) (Table 1 Treatment M). Subsequently, we designed another composition with sewage sludge filter cakes from 10 different sewage plants (AZV Leisnig, Leisnig, Germany) instead of manure to test another application of this method (Table 1 Treatments S).

**Table 1:** Composition of treatments 1 to 11.

Nr.	Treatment	Ingredients	Description
**1**	**M**	500 g manure from a dairy farm plus 94 g wheat bran, 38.5 g charcoal, 12 g sawdust, 61 g Bentonite clay, and 61.5 g basalt rock flour	basic manure treatment
**2**	**S**	500g different sewage sludge filter cake from 10 different plants 94 g wheat bran, 38.5 g charcoal, 12 g sawdust, 61 g Bentonite lay, and 61.5 g basalt rock flour	basic sludge treatment
**3**	**MEM**	384 g of treatment **M** 125 ml **EM** = “Effective Microorganism”	Basic manure treatment with additional *Lactobacillaceae* added
**4**	**M(EM)**_**a**_	384 g of treatment **M** 125 ml **a**utoclaved **EM**	control treatment for comparison with MEM to test the influence of additional *Lactobacillaceae*
**5**	**(MEM)**_**a**_	Treatment **MEM a**utoclaved	control treatment for comparison with MEM to test influence of the whole bacterial flora
**6**	**(M(EM)**_**a**_)_**a**_	Treatment **M(EM)**_**a**_ **a**utoclaved	control treatment to compare with M(EM)_a_ to test the influence of the whole bacterial flora
**7**	**S**_**Pool**_	Treatment **S**, but the filter cake was **pool**ed from 5 processing plants	pooled treatment S to reduce the sample number
**8**	**(S**_**Pool**_)_**a**_	Treatment **S**_**Pool**_ **a**utoclaved	control treatment to compare with S_Pool_, to test the influence of the indigenous fecal flora
**9**	**S**_**Pool**_-**WB**	Treatment **S**_**Pool**_ without (**−**) **w**heat **b**ran	sludge treatment S_Pool_ without wheat bran, to test the influence of the bran
**10**	**S**_**Pool**_-**WB+SD**	Treatment **S**_**Pool**_ without (**−**) **w**heat **b**ran but with (**+**) 36 g more **s**aw**d**ust to have comparable dry matter	treatment S_Pool_ with sawdust substituted (v/v) for wheat bran to provide comparable dry matter
**11**	**Fe+WB**	500 g **fe**ces plus 190g **w**heat **b**ran	reverse experiment to treatments S_Pool_-WB and S_Pool_-WB+SD

The final dry matter content should be around 20–40%

The “Lehr- und Versuchsgut Oberholtz” is a farm near Leipzig, integrated within the veterinarian studies and belongs to the veterinarian faculty of the University of Leipzig. Samples were taken during routine and/or education examinations, permitted by the administration (http://dekanat.vetmed.uni-leipzig.de/de/lvg).

The sewage sludge was delivered by the waste management company themselves (http://www.azv-leisnig.com/).

Following ingredients to the different faeces were added: wheat bran (LHG, Schmölln, Germany) to provide an easily digestible C-source to promote *Lactobacillaceae* growth and to decompose fodder residue; charcoal (Köhlerei, Tornau, Germany) as a non-rotting soil improver [24,27,28] for later use of the waste product as a fertilizer; sawdust (HVT, Germany, Dittersdorf), commonly used as litter in animal barns and as a low-digestible C-source within the subsequent fertilizer; Bentonite clay to provide nutrients (Beckmann and Brehm, Beckeln, Germany) and basalt rock flour (ABC-Baustoffe, Erfurt, Germany) as a mineral soil supplement [29] to promote the development of clay-humus complexes consistent with the archaeological findings.

The pooled filtrated sewage sludge had a pH of 6.2, a dry matter content of ∼ 11.9% containing of 9.4 organic matter and 2.5% inorganic matter. After adding the other components the dry matter content was 39,2% and comparable to the the dry matter of the manure matrix (∼ 40%) (Data kindly provided from Prüf- und Entwicklungsinstitut für Abwassertechnik an der RWTH Aachen e.V., Germany)

The initial concentration of lactate producing bacteria was compared with an optional *Lactobacillaceae* source called “Effective Microorganism” (EMa, Multikraft, Pichl/Wels, Austria) (treatments MEM and M(EM)_a_) and microbial activity of the indigenous faecal flora was investigated by autoclaving the mixtures (treatments (MEM)_a_ and (M(EM)_a_)_a_).

To identify the necessary components for the hygienisation process substitution experiments were carried out. Wheat bran was eliminated (treatment S_Pool_-WB) or substituted with sawdust, (treatment S_Pool_-WB+SD). Finally faeces and wheat bran only (Fe+WB) were investigated. Based on the results of treatment S_Pool_-WB and S_Pool_-WB+SD, enterococci, Gram-negative and yeasts were tested only.

### Bacteria, Viruses and Protozoa

Experiments were carried out with the following zoonotic microorganisms [30–34] *Salmonella* Senftenberg (DSMZ No. 10062), *Salmonella* Anatum (Lab No. 12602/2), *Listeria monocytogenes* (Lab No. 12602/5), *Staphylococcus aureus* (Lab No. 12602/4), *E*.*coli* O:157 (Lab No. 12602/3) and *Clostridium perfringens* (wild strain from manure). The effect of fermentation on spore forming pathogens was tested as well. Because of the close relatedness of *Clostridium sporogenes* (ATCC 3584) to *C*. *botulinum* A and F [35], we chose the less harmful *C*. *sporogenes* for these tests. In addition, enteric cytopathogenic bovine orphan virus (ECBO virus) and *Ascaris suum* eggs were evaluated to represent model pathogens with high environmental tenacity [36].

### Preparation of resistant mutants

A specially formulated agar for counting pathogens without interference from faecal flora. was developed using streptomycin and rifampicin resistant strains of *Salmonella* Anatum, *Listeria monocytogenes*, *E*.*coli* O:157 and *Staphylococcus aureus* according to Linde [37,38]. Single antibiotic-resistant mutants were selected by streaking approximately 10^10^ cfu of fresh bacterial mass on nutrient agar 1 (Sifin, Germany, Berlin) supplemented with 400 mg streptomycin (Roth, Germany, Karlsruhe) and 300 mg rifampicin (Infecto Pharm, Germany, Heppenheim) per litre, incubating the plates aerobically at 37°C for 24 h, picking individual colonies from the plate and subculturing them twice in antibiotic supplemented nutrient broth.

### Community analyses

The following culture media were used for bacterial counts: Columbia blood agar (Oxoid, Germany, Wesel) for aerobic and anaerobic organisms, Water-blue-Metachrome-yellow Lactose agar (acc. to Gassner, Sifin) for Gram-negative organisms, Citrate-Azide-Tween-Carbonate Agar (CATC, Sifin) for enterococci, Sabouraud-Agar (Oxoid) for yeasts and moulds, Neomycin Polymyxin blood agar [NeoP, neomycin 200 mg/l (Roth, Germany, Karlsruhe), polymyxin B 100 mg/l (Fluka, Switzerland, Basel), blood 10% (cattle), glucose 5g/l and nutrient agar 1 (Sifin)] for Clostridia and MRS Agar (Oxoid) for lactic acid bacteria. Bacterial counts were performed using the plate count method and the most probable number (MPN) [39] for sulphite-reducing, spore-forming bacteria (SRSF). To achieve this, the samples were heated for 10 minutes at 80°C [40,41], diluted by a factor of 10 and cultivated in liquid differential reinforced Clostridia medium (DRCM, Oxoid). Pasteurization at 80°C guaranteed death of enterococci which survived a 60°C treatment. The isolated colonies were analyzed with MALDI-TOF MS (Bruker, USA, Billerica) as described by Shehata [42].

### Inactivation of bacterial pathogens

To test inactivation of pathogenic bacteria, 3 ml suspensions (10^7^ to 10^9^ cfu/ml) of *E*.*coli* O:157, *S*. Anatum, *Listeria monocytogenes*, *Clostridium (C*.) *sporogenes or C*. *perfringens* were added to 27 g of treatments MEM and M(EM)_a_. To test the influence of the native bacterial flora, we autoclaved control samples before adding the pathogen suspension (treatments (MEM)_a_ and (M(EM)_a_)_a_). We incubated samples under anaerobic conditions (Anaerocult, Merck) at 37°C for 21 days. The concentration of the spiked pathogens was determined at 0, 3, 7, 14 and 21 days incubation by taking 0.5 g of the treatment, diluting it in 4.5 ml PBS and plating on culture media (as described above) and also nutrient agar 1 (Sifin) supplemented with 400 mg streptomycin, 300 mg rifampicin and 1 g glucose per l. The detection limit was set at 100 cfu/g with the plate count method and approximately 10–100 cfu/g in the direct smear and in the *Salmonella* enrichment (acc. to Preuss, Merck).

To simulate waste containing several pathogens, we prepared four pathogen mixtures. Preliminary experiments had previously shown inactivation of non-sporulated pathogens (unpublished data):

**Mix 1** (*Salmonella* Anatum, *Listeria monocytogenes*, *E*.*coli* O:157, and *C*. *perfringens*),
**Mix 2** (*Salmonella* Senftenberg, *Staphylococcus aureus*, *C*. *sporogenes*)
**Mix 3** (*Salmonella* Senftenberg, *Listeria monocytogenes*, *Staphylococcus aureus*, *E*.*coli* O:157, *C*. *perfringens*, *C*. *sporogenes*)
**Mix 4** (autoclaved cultivation broth).

We designed Mixes 1 to 4 to facilitate rapid differentiation on the plates. Because *Salmonella* serotypes are not differentiable on a plate, they were evaluated separately. In addition to *S*. Anatum, the heat-tolerant *Salmonella* Senftenberg [43] were tested since it is a common serotype in waste treatment processes [44, 45]. From the experience with *S*. Anatum, we did not modify *S*. Senftenberg for antibiotic resistance, because of the well-working selective gassner media. In order to keep the concentration of each pathogen in the treatment group as high as possible, mix 1 and 2 contained only three to four pathogens, whereas control mix 3 contained six. The initial inoculum was from fresh (overnight) cultures so that the final concentration depended on the specific generation time in nutrient broth I (Sifin) or RCM-broth (Sifin) for Clostridia. After gently mixing 3 samples per treatment, we counted them before and after 3 days of fermentation.

Mix 1 and 2 were added to the non-autoclaved treatments while mix 3 was added to the autoclaved treatments. We added Mix 4 to the non-autoclaved treatments as a control sample to investigate VFA development without additional bacteria. The rate of sporulation of *C*. *perfringens* and *C*. *sporogenes* were analyzed by counting the inocula before and after heating at 80°C for 10 minutes by the MPN method. Based on the experiences with manure treatments MEM, M(EM)_a_, (MEM)_a,_ (M(EM)_a_)_a_ and the high natural *C*. *perfringens* concentration within the sludge, the experimental design of treatments S_Pool_ and (S_Pool_)_a_ were simplified in order to test the disinfection effect of treatments 7 & 8 S_Pool_ and (S_Pool_)_a_ with non-spore forming bacteria only. We counted spiked samples before and after 3 days of incubation.

### Weight loss during fermentation

We measured mass loss during sludge-fermentation in 50 ml tubes by weighing before and after 21 days of fermentation and subtracting the difference from the initial weight (27 g). This data was compared with compost mass loss reported in the literature.

### pH Value

Since bactericidal effectiveness of VFA depends on pH, the pH were measured by placing 5 ml of the sample in 20 ml KCl—solution (1 mol/l), shaking for 60 minutes, and then measuring the pH with a pH-meter (GPH14 Greisinger electronic, Germany, Regenstauf) according to DIN ISO 10390.

### Volatile fatty acids (VFA), lactate and alcohol analyses

During the bacterial inactivation process of treatments MEM, M(EM)_a_, (MEM)_a_ and (M(EM)_a_)_a_, we measured acetic acid, propionic acid, i- and n-butyric acid, i- and n-valeric acid, n-caproic acid, ethanol, propanol, butanol, 2,3-butanediol, and 1,2-propanediol concentrations, according to VDLUFA [36]. Lactic acid concentration were analyzed according to Haacker [46]. The mean value from 3 samples per treatment before and after 3 days of fermentation were calculated. Based on the information obtained with one sampling for experiments with treatments 3–6, we added several time slots while monitoring treatment S_Pool_. The VFA observations of treatment S_Pool_ were made without spiking the samples with additional pathogens (3 samples each).

### Inactivation of ECBO

The non-coated bovine enterovirus (BEV), also known as enteric cytopathogenic bovine orphan virus (ECBO), is commonly used for testing disinfection products due to its high tenacity. Pre-swollen 1 cm² x 1 mm lime-wood platelets were incubated for one hour with an ECBO virus (LCR-4) suspension and added it to treatment MEM and M(EM)_a_. (acc. to DVG, [47]. After mixing the matrices with 3 platelets per tube, treatments MEM and M(EM)_a_ were incubated at 37°C. The virus titre was measured directly after mixing and also after 3, 7, 14 and 21 days by 4-MPN-rows on foetal calf lung cells. Different starting concentrations of lactic acid cultures were tested (treatments MEM, M(EM)_a_).

### VFA- Bioassay experiments

Bioassay experiments with *S*. *aureus* tested the influence of single VFA at ph 5.3 as well as a VFA mixture with the same concentration as the 4 most highly concentrated VFAs found in this study (Table 2) and 0.15% ethanol (Roth) at pH 5.3 (adjusted with NaOH). The VFA mixture consisted of nutrient broth I (Sifin), lactic acid 1% (Roth), acetic acid 0.46% (Roth), n-butyric acid 0.12% (Roth), propionic acid 0.1% (Merck) and i-butyric acid 0.01% (Alfa Aesar, USA, Wardhill, MA) as the single VFA at the same concentration. The test tubes containing 5 ml broth were inoculated with 10 μl *S*. *aureus* suspension (6.15 * 10^5^ cfu/ml) and incubated them at 37°C for 3 days.

**Table 2:** Concentration (g/kg fresh weight) of VFA and lactic acid, in treatment S_Pool_ during the fermentation process.

Treatment (time)	acetic acid	propionic acid	i- butyric acid	n- butyric acid	i- valeric acid	n- valeric acid	lactic acid
**S**_**Pool**_ (start)	0.86	1.32	0.07	0.11	0.09	0.07	4.90
**S**_**Pool**_ (20 h)	3.82	1.12	0.07	1.25	0.08	0.07	9.83
**S**_**Pool**_ (47 h 15’)	4.17	1.08	0.07	1.19	0.08	0.07	12.17
**S**_**Pool**_ (3d 16h)	4.60	1.03	0.06	1.17	0.08	0.06	12.47

### Inactivation of *Ascaris suum*

The viability of *Ascaris suum* eggs in treatments MEM and M(EM)_a_ was tested by adding 3 ml of a tap water suspension containing 27,000 eggs per ml to 27 g of the matrix in tap water and gently mixing. We tested the influence of different starting concentrations of lactic acid cultures (treatments MEM, M(EM)_a_) after 0, 7, 21 and 56 days, respectively, by taking three samples per time slot and treatment. *Ascaris* eggs were isolated from the manure matrices by flotation, re-suspending in tap water, transferring to 6 well tissue culture plates (TPP) and incubating at 25°C for 20 days with aeration every second day. To determine the rate of embryonation, 300 eggs were analyzed from each sample (acc. to DVG). Since ascariasis does not occur in cattle in northern Europe, the matrices were not investigated for eggs before spiking.

## Results

### Community analyses

**Fig 3:**
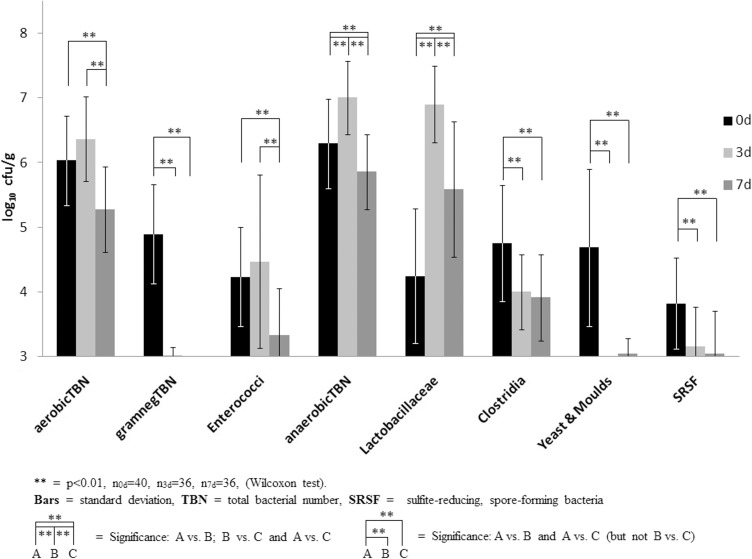
Typical bacterial community shift during fermentation of treatment S.

The mean values and standard deviations of the bacterial counts of sewage sludge matrices (treatment S) from 10 different decentralized sewage plants are shown in Fig. 3. The typical faecal flora before fermentation was changed dramatically after fermentation. A general overview of a typical community shift in all of the tested faecal treatments is presented in Table 3. Species relevance during fermentation of treatment S and M is shown in the S1 Appendix. Similar community shifts also occurred in medium—scale experiments (1 and 60 litre containers) and also at lower incubation temperatures (27°C, data not shown), but took a few days longer at the lower temperature. Furthermore, these experiments showed that this method is even easier to handle on a larger scale. Because anaerobic conditions are self establishing as long as the matrix is packed well, the air column within the bowl is kept small (approximately <10–5%) and a fermentation lock is installed. A small air column is needed because the material swells and may plug the lock.

**Table 3:** General overview of a typical community shift (cfu/g).

Group	Start	3 d fermentation	7 d fermentation
Gram-positive, non-spore-forming (Lactobacillaceae, Enterococci excluded)	10^6^–10^8^	< 10^2^	< 10^2^
Bacillaceae	10^3^–10^5^	10^3^–10^5^	10^3^–10^5^
Gram-negative	10^5^–10^7^	< 10^2^	< 10^2^
Enterococci	10^3^–10^6^	10^3^–10^6^	< 10^3^–10^4^
Lactobacillaceae	10^3^–10^4^	10^5^–10^7^	10^3^–10^6^
Yeasts / Molds	10^3^–10^4^	< 10^2^	< 10^2^
Clostridiaceae	10^3^–10^6^	10^3^–10^5^	10^3^–10^6^

The smell of the treatments changed from a typical faecal smell to a sour, silage-like odour with fermentation. Gram-negative bacteria, enterococci or yeasts and moulds could not be found in treatments S and Fe+WB, but were present in treatments without the wheat bran (S_Pool_-WB and S_Pool_-WB+SD, Table 4).

**Table 4:** Community shifts within treatments S, S_Pool_-WB, S_Pool_-WB+SD, Fe+WB.

Group	start	3 d fermentation	7 d fermentation					
Treatment:	S, S_pool_-WB, S_Pool_-WB+SD	S	S_Pool_-WB	S_Pool_-WB+SD	S	S_Pool_-WB	S_Pool_-WB+SD	Fe+WB
Gram-positive, non-spore-forming (Lactobacillaceae, Enterococci excluded)	10^6^	< 10^2^	10^4^	10^4^	< 10^2^	10^4^	10^4^	nt.
Bacillaceae	10^4^	10^4^	10^4^	10^4^	10^4^	10^4^	10^4^	nt.
Gram-negative	10^5^	< 10^2^	10^5^	10^6^	< 10^2^	10^4^	10^6^	< 10^2^
Enterococci	10^6^	10^4^	10^7^	10^7^	< 10^2^	10^5^	10^6^	< 10^2^
Lactobacillaceae	10^6^	10^7^	10^7^	10^7^	10^6^	10^5^	10^4^	nt.
Yeasts and Moulds	10^4^	< 10^2^	10^4^	10^4^	< 10^2^	< 10^2^	< 10^2^	< 10^2^
Clostridiaceae	10^6^	10^5^	10^6^	10^6^	10^6^	10^6^	10^6^	nt.

nt. = not tested

### Inactivation of bacterial pathogens

**Fig 4:**
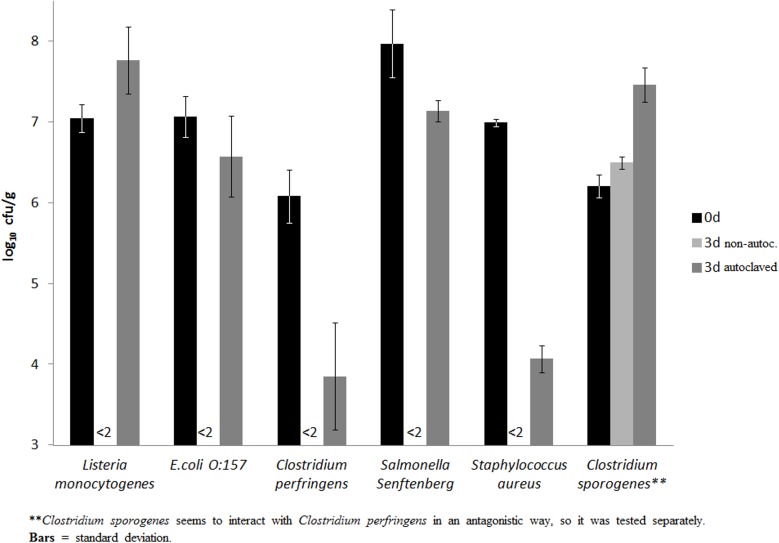
Development of bacterial pathogens in treatments MEM and (MEM)_a_.

All non-sporulated pathogens where inactivated in the non-autoclaved treatments (treatment MEM, Fig. 4), but sporulated pathogens survived fermentation. *Listeria monocytogenes* and *C*. *sporogenes* grew in autoclaved treatment 5, while the concentration of the other pathogens decreased but still remained above the minimum detection level. There were only slight differences between treatments M(EM)_a_ and (M(EM)_a_)_a_ compared to 3 and 5. Also the sewage sludge data did not change. The hygienisation ability of non-autoclaved sludge (treatment S) was the same as with non-autoclaved cow manure (treatments M, MEM and M(EM)_a_).

### Weight loss

After 21 days of fermentation, the mean weight loss of the sludge treatment (S) was 0.66 g (standard deviation = 0.052 g) which was 2.44% of the total mass of 27 g (p = 0.031 n = 6, Wilcoxon test).

### pH Value

The pH decreased in each group independent of treatment during the 3 days fermentation (Table 6) with only the autoclaved controls staying at nearly the same pH. The pH of the non-autoclaved treatments decreased to a much lower level than the autoclaved treatments. Treatment MEM dropped the pH lower than treatment M(EM)_a_. The drop in pH of treatment S was similar to the unspiked treatment M(EM)_a_ that dropped to 5.3.

### Volatile fatty acids (VFA), lactate and alcohols

The concentration of VFA and alcohols (g/kg) in the different manure treatments spiked with bacterial pathogens (treatments MEM, M(EM)_a_, (MEM)_a_ and (M(EM)_a_)_a_) during the hygienisation process are shown in Table 5. N-valeric acid, n-caproic acid, butanol, 2,3-butanediol, 1,2-propanediol and lactate were below the detection limit of 0.05 g/kg and 0.1 g/kg for lactic acid. After 3 days, the highest concentration of n-butyric acid (93 mM) was found in treatment MEM, mix 1 alone with 28 mM acetic acid and a pH of 5.50. The lowest concentration of n-butyric acid (5 mM) was in treatment MEM, mix 4 which also had 47 mM acetic acid and a pH of 4.94.

**Table 5:** Concentration (g / kg fresh weight) of VFA and alcohols in treatments MEM, M(EM)_a_, (MEM)_a_ and (M(EM)_a_)_a_ at the start and after 3 days fermentation.

Treatment (time in days)	Bacteria Mix	acetic acid	propionic acid	i- butyric acid	n- butyric acid	ethanol	propanol	i- valeric acid
**(M(EM)**_**a**_)_**a**_ **(0d)**	**Mix 4**	1.22	0.29	0.06	0.08	< 0.05	< 0.05	< 0.05
**(MEM)**_**a**_ **(0d)**	**Mix 4**	1.35	0.28	0.05	0.09	< 0.05	< 0.05	< 0.05
**(M(EM)**_**a**_)_**a**_ **(3d)**	**Mix 4**	1.13	0.26	0.05	0.07	< 0.05	< 0.05	< 0.05
**(MEM)**_**a**_ **(3d)**	**Mix 4**	1.33	0.27	0.05	0.09	< 0.05	< 0.05	< 0.05
**(M(EM)**_**a**_)_**a**_ **(3d)**	**Mix 3**	2.17	0.24	0.09	0.55	0.80	0.11	0.11
**(MEM)**_**a**_ **(3d)**	**Mix 3**	2.06	0.23	0.09	0.47	0.76	0.11	0.11
**M(EM)**_**a**_ **(0d)**	**Mix 4**	2.20	0.20	0.06	0.07	1.85	< 0.05	< 0.05
**MEM (0d)**	**Mix 4**	2.79	0.26	0.06	0.09	1.99	< 0.05	< 0.05
**M(EM)**_**a**_ **(3d)**	**Mix 4**	1.68	0.26	0.05	4.42	1.28	0.04	< 0.05
**MEM (3d)**	**Mix 4**	2.85	0.25	0.05	0.46	1.33	0.14	< 0.05
**M(EM)**_**a**_ **(3d)**	**Mix 1**	2.63	0.25	0.05	4.30	1.55	0.14	< 0.05
**MEM (3d)**	**Mix 1**	1.67	0.27	0.05	8.23	1.45	0.13	< 0.05
**M(EM)**_**a**_ **(3d)**	**Mix 1**	1.92	0.28	0.10	4.21	1.29	< 0.05	0.12
**MEM Mix 2 (3d)**	**Mix 1**	3.14	0.29	0.10	2.13	1.34	< 0.05	0.12

Shifts in VFA and lactic acid (g/kg) in a pooled sample of treatment S_Pool_ during fermentation are shown in Table 2. While several materials stayed at the same concentration, acetic acid and n-butyric acid increased, and propionic acid decreased. In contrast to the non-measurable lactic acid concentrations (Table 5) Lactic acid increased to 1.25% (wt/wt) in treatment S_Pool_ (Table 2).

### Bioassay experiments

Most of the VFAs slightly inhibit growth of *S*. *aureus* but were not bactericidal at this pH and concentration. Although the most substantial, nearly bacteriostatic, effect of the mixture was caused by acetic acid, a combination of all VFAs plus ethanol tended to be more effective than acetic acid alone. Lactic acid alone at pH 5.3 appeared to support the growth of *S*. *aureus*.

### Inactivation of the ECBO virus

Active ECBO virus was reduced from 5.49 log_10_ cpe/ ml to below the detection limit of 3 log_10_ cpe/ml within 14 and 7 days fermentation of non-autoclaved manure treatments MEM and M(EM)_a_, respectively, either with or without EM supplementation in starter cultures.

### Inactivation of *Ascaris suum*

There was no reduction in the rate of embryonation of *Ascaris suum* eggs during fermentation of non-autoclaved manure (treatment MEM, 96.8%, SD: 1,90% and treatment M(EM)_a,_ 97.1%, SD: 2,10%) within the first 3 weeks; however, after 56 days of fermentation, embryonation dropped to 0%. A similar drop was observed in tap water after 56 days of anaerobic incubation at 37° C.

## Discussion

### Community shift

**Fig 5:**
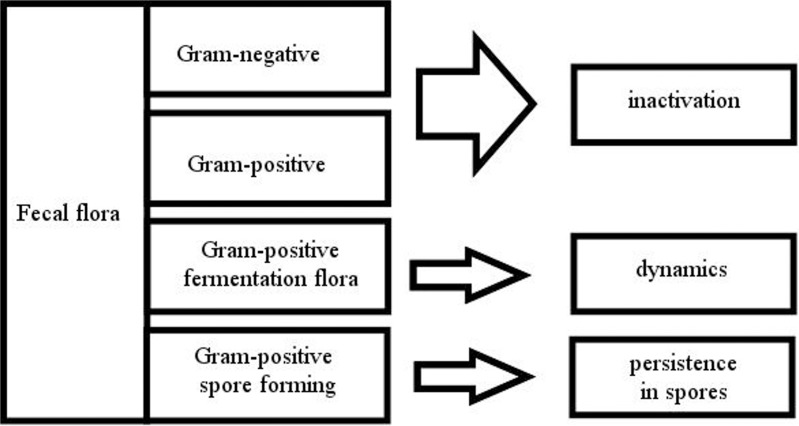
Flow chart of a typical community shift during fermentation.

As shown in Fig. 5 and in detail in the apendix massive changes within the bacterial flora were observed. All Gram-negative bacteria and yeasts decreased to levels beneath the detection limit after 3 days of fermentation. Changes in the concentration of Enterococci and Lactobacilli were striking within the first 7 days. This system seems to be specific for certain groups or shifts too fast to be effectively observed at the chosen points of time. Additional experiments showed that Enterococci were not detectable after 21 days (data not shown). The starting concentration of Lactobacilli and Enterococci probably will have a major influence. All non-spore-forming bacteria except acid-tolerant organisms like *Leuconostoc mesenteroides*, were inactivated beneath the detection limit. Spore-forming bacteria such as *B*. *licheniformis*, *B*. *cereus* or *Paenibacillus sp*. survived fermentation. It is interesting that a standardized flora was not detected with the methods used. *Pediococcus pentosaceus* was found frequently in treatment S, although not in all samples. Different species may be competing for similar ecological niches. Similarities within Lactobacilli, Enterococci, coliforms and pH shifts could be found within Hrubants work with fermentation of feedlot waste, but the yeast shift data they observed is completely different [48].

Elimination and substitution experiments showed that the presence of an easily digestible C-source like wheat bran is essential for the inactivation of Gram-negative bacteria and suppression of *Clostridia*. The developing flora without wheat bran was very different from those with wheat bran. Neither Charcoal nor any other additive except wheat bran was necessary for hygienisation, but might improve the soil by absorbing toxins, stabilizing nutrients and forming clay-mould-complexes [24,29,49].

*Enterococci* should be the preferred microorganism for qualitative tests of successful bacterial hygienisation of fermented faecal matrices because of their obligatory presence in tested faecal matrices and their higher tenacity compared to Enterobacteria. The sour silage-like smell of fermented matrices could be a no-tech indicator of successful fermentation by an experienced user of the method.

### Hygienisation of bacterial pathogens

Since all added non-sporulated pathogens died in the non-autoclaved treatments, this hygienisation method seems to be much more effective than composting [11,50,51] because all Gram-negative and Enterococci were inactivated within a few days. So the reaction of the added pathogens was quite the same as of the inherent flora. Gram-positive and Gram-negative bacteria were inactivated whether they were unsporulated or not belonging to the *Lactobacillaceae*. It also is more effective than 60°C/1h pasteurization within biogas plants [10,44], because all Enterococci were inactivated and re-colonisation of gram-negative or Enterococci was never observed during storage. This is probably because the environment is more stable as long as it is not aerated. There is no need for additional technical equipment such as pasteurisation units or compost-turners with this hygeinization process. Every farmer who produces silage will be able to ‘ensilage’ manure. The effective inactivation of *C*. *perfringens* may be attributed to the fact that this pathogen did not sporulate (< 10 cfu/ml) in the RCM-media (Oxoid) or other matrices tested. In contrast, the inactivation of *C*. *perfringens* and *S*. *aureus* in the autoclaved treatment (MEM)_a_ may have been caused by interactions with the other organisms of mix 3. Another example of pathogen interactions was that *C*. *sporogenes* was not even detectable after incubation with *C*. *perfringens* in treatment (MEM)_a_. Because of this potential interaction, *C*. *sporogenes* was tested separately and then grown in treatment (MEM)_a_. In contrast to the minor sporulation of *C*. *perfringens*, about 50% of *C*. *sporogenes* cells sporulated and were not inactivated in non-autoclaved or autoclaved matrices. The growth of *C*. *sporogenes* demonstrate what may happen in the absence of the inherent antagonistic flora. Moreover a slight growth occurred even within the non autoclaved flora. This might be discussed as a result out of the inaccuracy of the plate count method, but it was sporadically observed in other inherent clostridia grow-cases in matrix S as well. The mean concentration of Clostridia sunk strongly and significantly p < 0.01 as shown in Fig. 3, but nevertheless a growth occurred in 6 of 37 cases. It is not clear why the growth occurred but we suspect that the wheat bran with its high amount of proteins (∼15% [52]) may be a suitable resource for Clostridia. Therefore and due to the fact that wheat bran is an edible product we strongly suggest a nother carbon source e.g. green waste ore else.

The slight difference of ∼0.3 log_10_ between the start and the 3^rd^ day of fermentation of *C*. *sporogenes* in the non-autoclaved matrices (treatments MEM & M(EM)_a_) is not statistically different with the colony count method. The SRSF and Clostridia numbers dropped significantly (∼0.65 log_10_ resp. ∼0.75 log_10_) within the first 3 days of fermentation in sludge matrices (treatment S, Fig. 3). There might be some ingredients or detergents which facilitate the inactivation of spores in the sludge. Since indigenous sludge-borne *C*. *perfringens* was not strongly inactivated, it indicates they were sporulated. Sporulated pathogens which survived the 80°C heat treatment before counting, will obviously survive 60°C during composting or pasteurisation also. The method used in this study reduced pathogens more effectively than the fermentation and vermicomposting reported by Factura [53].

It was not determined whether heat sterilization of the control samples (which surely affected the matrix) improved or decreased the growing conditions for the tested pathogens. Gamma sterilization may have been a more gentle treatment for the control samples and should be tested. Nevertheless, the substitution experiments with treatments S_Pool_, S_Pool_-WB, S_Pool_-WB+SD and Fe+WB show the need for the addition of wheat bran to direct development of the original faecal flora.

Although concentrations of the initial infectious pathogens were high when tested individually (data not shown), the concentrations ranged from 3 to 6 times higher because they did not get diluted by the other pathogens in the mix. Independent of the concentration, they were inactivated within 3 days. The main focus of this work was the inactivation of fresh, highly concentrationed pathogens to well below their detection limit. It did not evaluate individual effects occurring between the various microbes.

We recognize that the use of mutants may result in reduced environmental tolerance of the microorganism; however, Linde [54] reported the opposite is possible as well. Linde described mutants dying more slowly because of their slower metabolism, which caused an indirect proliferation in harsh environments. The mutant pathogens we used were tested after 24 hours of incubation in a hypotonic solution which should have improved their stress tolerance by stabilizing their cell walls with oligosaccharides [55]. Even with this treatment, they were inactivated within 3 days along with all of the other added or inhabiting (opportunistic) pathogens that were excreted by cattle or humans.

### Loss of weight during fermentation

The 2.44% weight loss in the first 21 days was smaller than the mass loss with composting which may vary from 15% [56] to 57% [12] after 42 and 132 days respectively. In general, composting time periods are longer than 21 days, but the highest emissions of CO_2_ [57], NH_3_ and heat [13] are generally observed in the first 21 days of composting to indicate that the loss of mass probably is also highest in the first 3 weeks. Although the anaerobic pots (2.4 dm³) are closed systems, gas production of the matrices was small enough that there was no detectable pressure when opening the pots. Instead, the caps on the pots did not lift by themselves after unlocking the clamps. Medium-scale experiments with gas traps and a gas analysis could give more information about mass flows within the matrices.

### pH value

Only the control group treatment MEM, mix 4 reduced the pH below 5 as shown in Table 6. Most non-autoclaved matrices with pathogens dropped to a pH of around 5.5 to 5.2. Those values alone do not explain the hygienisation effect since *Salmonella* and *E*. *coli* can grow at pH 4.5 [58] and *S*. *aureus* survives at pH 2.5 [20].

**Table 6:** pH of treatments MEM, M(EM)_a_, (MEM)_a_ and (M(EM)_a_)_a_ at the start and after 3 days fermentation with different pathogen mixes.

Treatment	Bacteria Mix	0 d	3 d
**(M(EM)**_**a**_)_**a**_	broth only	6,72 (0,05)	6,70 (0,09)
**(MEM)**_**a**_	broth only	6,70 (0,02)	6,63 (0,04)
**M(EM)**_**a**_	broth only	6,68 (0,04)	5,37 (0,17)
**MEM**	broth only	6,70 (0,04)	4,94 (0,02)
**MEM**	Mix 1	n.a.	5,50 (0,03)
**M(EM)**_**a**_	Mix 1	n.a.	5,64 (0,10)
**MEM**	Mix 2	n.a.	5,10 (0,17)
**M(EM)**_**a**_	Mix 2	n.a.	5,55 (0,04)
**(MEM)**_**a**_	Mix 3	n.a.	6,08 (0,02)
**(M(EM)**_**a**_)_**a**_	Mix 3	n.a.	6,10 (0,03)

(Standard deviation).

### Volatile fatty acids

VFA become bactericidal at a certain pH [22]. The chemical conditions we measured were comparable to the reports of Presser [21] and Knarreborg [19] who showed that bactericidal concentrations were not present and, therefore, could not explain a total loss of 10^8^ pathogens within 3 days. Knarreborg reported that it takes 100 mM of butyric acid at a pH of 5.5 and 37°C under anaerobic conditions in a gut simulator to reduce coliform bacteria by 6% per hour. This concentration was not reached in our study, but even if this had happened, the residual coliforms would not have fallen below 10^6^ cfu after 72h.

The higher concentration of lactic acid during fermentation of treatment S_Pool_ may have been caused by the higher dry matter content since 125 ml of EM suspension was not added. The measured concentration of 138.5 mM/l and pH of 5.3 are still adequate growing conditions for *E*.*coli* according to Presser and Knarreborg [19,21].

Goepfert [18] reported the greatest bactericidal effect of acetic acid, and lesser effects of propionic acid and butyric acid to *S*. *typhimurium* at 0.5%, with exposure periods ranging from several hours to 2 days at 37°C and a pH of 5.0–5.5. Lind [20] described a bacteristatic effect of 0.3% acetic acid or 0.5% lactic acid on the extremely pH tolerant *Staphylococcus aureus* within 2 days. In contrast, it took 0.5% and 1.0%, respectively, for a bactericidal effect within 24h in the presence of proteins within a pH range of 2.5 to 3.5. In summary, we found > 0,5% VFA after 3 days in each measured group except for the group in treatment MEM, mix 4. The pH in this group was the only one lower than pH 5 which increases the bactericidal effectiveness of VFA. In addition, there seems to be a synergistic effect with a mixture of VFA and a low alcohol concentration as observed with *S*. *aureus*, although this mixture was bacteriostatic rather than bactericidal.

If pH, in combination with the measured fermentic acid concentrations, are as efficient in our matrices (similar to what Goepfert described within aqueos solution), they could be assumed to be the reason for the hygienisation observed. Even if they are not solely responsible for the succesful disinfection, other factors made them effective, e. g. adsorbtion of higher VFA concentrations by charcoal. Various concentrations of VFA are reported to have an effect, but were not measurable in our study after adsorbtion by charcoal or the development of a VFA-digesting bacterial flora. The bacteriostatic, but not bactericidal, effects of VFAs measured in bioassay experiments indicate that unknown interactions or other chemical parameters could be involved. Perhaps bacterial interactions involving the production of bacteriocins, e.g. pediocin of *Pediococcus pentosaceus*, could be responsible for inactivation of the non-spored bacteria. Many species of known bacteriocin-forming bacteria beside *P*. *pentosaceus* were found in the matrices during the fermentation process. Non-culturable bacteria also may play a role and should be investigated by T/DGGE or metagenomic analysis and rtPCR for example.

### Inactivation of ECBO

This virus tends to be inactivated about 4 days earlier in treatment M(EM)_a_ than in MEM, which indicates an influence of the matrix. Boegel [59] reported that BEV decreased 0.5 log_10_ per day at 37°C and that it survived better at lower temperatures. Nazir [60] showed that the tenacity of the ECBO virus depends strongly on both temperature and matrix effects. Similarly, Lund [61] reported a 4 log_10_ reduction of BEV at 35°C in physiological saline within 219 hours or within 23 hours in manure containing bleaching clay under anaerobic conditions at pH 8.0. In contrast, Biermann [62] reported very strong temperature stability of the ECBO virus, which lost only 2 log_10_ infectious units in 26 weeks at 20°C in liquid cow manure. The effect of autoclaving was not tested in this study because of the obvious need of non-autoclaved treatments to inactivate the obligatory bacteria in faeces. More investigations should be performed in the future to discover the matrix and temperature effects on inactivation of the ECBO virus in fermentation processes.

### Inactivation of *Ascaris suum*

*Ascaris suum* eggs remained generally unaffected by incubation the first 21 days. The loss of pathogenicity of *A*. *suum* eggs between 21 and 56 days, as well as in the tubes spiked with EM (MEM) and control tubes (M(EM)_a_) and tap water tubes will probably be attributed to the 37°C temperature. Earlier studies with eggs of the related species, *A*. *lumbricoides*, showed that a temperature of 37°C over a long period is harmful to eggs [63]. Seamster [64] reported the optimum temperature for embryonation of *Ascaris suum* eggs was 31.3°C and that development slowed down as the temperature increased. Only 10% of the eggs developed a motile embryo at 34.4°C, and all ova died in early cleavage stages between 35.6 and 37.8°C. In a mesophilic anaerobic digester maintained at 35°C, the viability of *A*. *suum* eggs dropped from 95% after one week to 50% after 5 weeks [65]. Even the development at 32°C appeared to be suboptimal since more than 50% of the infective eggs showed a gradual decline in larval motility and healthy protoplasmic appearance [66]. Maya [67] also reported that the viability of *Ascaris* eggs stored in sludge under various conditions decreased as the temperature increased. Storage temperature was found to be the most important factor affecting the viability of *Ascaris* eggs in sludge [68]. Other factors, such as type of sludge digestion, storage in soil versus sludge, pH and egg species had only minor effects. Thus, the influence of fermentation on *A*. *suum* eggs seems to be minor under the investigated conditions even though the core temperatures of 60–70°C that develop during composting are high enough to kill those eggs within several hours [12,63]. Nevertheless, the survival of *Ascaris sp*. during composting for several months has been reported previously [51], but this may result from different temperature zones in compost piles. There is an obvious need to test the fermentation method under field conditions since daily temperature shifts or heating (e. g. storing the fermented product in black barrels in direct sunlight) could overcome the limitation in hygienisation of parasite eggs during fermentation. The combined positive aspects of heat from composting and the effects of fermentation should be tested also. There were concerns about the effectiveness of temperature on bacterial pathogens but, as shown in these experiments, the C-source and living faecal flora seem to provide the predominant influence on bacterial disinfection.

## Conclusion

The lactic acid fermentation is a hygienisation method which may be comparable to a pasteurization in the result except for the survival of the lactic acid bacteria. It is a stand-alone possibility for inactivation of pathogens independent of a further use as fertilizer or further biogas production, which is possible as well, as shown by Herrmann [69].

Fermentation was able to hygienise faecal waste from small-scale sludge plants and animal farms much more effectively and faster than composting. Since the lack of self-heating and with only a small amount of gasproduction, this method also conserves nutrients during processing much more effectively than aerobic composting methods [12].

The conserved nutrients could be used to promote a more efficient agriculture. All in all, fermentation seems to be a suitable, fast, easy-to-use, low-tech method to break viral and bacterial infection cycles by producing a hygienized organic fertilizer within 7 days. This study does not support a need for additional microorganisms, such as the tested EM, as long as a living fecal microbiota and a fermentable substrate are present.

The principle of how hygienisation works is not completely clear, although VFA and temperature were shown to have an important influence on bacterial survival. These experiments showed that bacterial hygienisation also worked at 27°C. Further investigation, especially at lower temperatures (21°C, 15°C and 5°C) are necessary to test fermentation benefits under field conditions.

## Supporting Information

S1 Appendix List of species of treatment M and S. (DOC)
