# Supplementary material for: Hygienisation and Nutrient Conservation of Sewage Sludge or Cattle Manure by Lactic Acid Fermentation
Source: PLoS One. 2015 Mar 18;10(3):e0118230. doi: 10.1371/journal.pone.0118230 (PMC4364889; doi:10.1371/journal.pone.0118230)
Supplement: S1 Appendix — (DOC) [file pone.0118230.s001.doc]

# Evaluation of species of matrix M

List of Gram-negative microorganisms of matrix **M**, their relevance and status during or after fermentation. Green: positive effect blue: neutral effect red: negative effect

| **Species** | **Evaluation** | **Significance** | **During / After Fermentation** |
| --- | --- | --- | --- |
| *Enterobacter amnigenus* | Potential pathogen [1] | Sometimes clinically relevant | Inactivation |
| *Escherichia coli* | Intestinal flora, pathogen [2], | Highly clinically relevant | Inactivation |
| *Pantoea agglomerans* | Human- and phyto pathogen [3] | Clinically relevant | Inactivation |
| *Providencia rettgeri* | Human- und insect pathogen [4,5] | Clinically relevant | Inactivation |
| *Pseudomonas flavescens* | Plantassoziated environmental microorganism (MO) [6], genus contains pathogens as well [7] | Unknown, nonpathogenic, possible model organism | Inactivation |

List of Gram-positve spore forming microorganisms of matrix **M**, their relevance and status during or after fermentation. Green: positive effect blue: neutral effect red: negative effect

| **Species** | **Evaluation** | **Significance** | **During / After Fermentation** |
| --- | --- | --- | --- |
| *B. licheniformis* | Environmental MO , uncommon food spoiler [8] [9], genus contains pathogens [10] [11] | Unknown, not clinically relevant [12], possible model organism | Survivial in spore |
| *B. pumilus* | Soil MO, supports plant growth | Antagonice phytopathogenes [13] | Survivial in spore |
| *Brevibacillus agri* | Doubtful pathogen [14] [15], environmental MO [16–18] | Unknown clinical relevance [19] | Survival in spore |

List of Gram-positve microorganisms of matrix **M**, their relevance and status during or after fermentation. Green: positive effect blue: neutral effect red: negative effect

| **Species** | **Evaluation** | **Significance** | **During / After Fermentation** |
| --- | --- | --- | --- |
| *Aerococcus viridans* | Potential pathogen [20] | Sometimes clinically relevant | Inactivation |
| *Bifidobacterium pseudolongum* | Probiotic [21] [22] | Economically relevant | Inactivation |
| *Corynebacterium casei* | Cheese production [23] , genus contains pathogens as well [24] | Economically relevant nonpathogen, possible model organism | Inactivation |
| *Enterococcus faecium* | Commensal flora and pathogen [25], less virulent than *E. faecalis* [26] [27], bactericide and bacteriocin former [28] [29] | Highly clinicaly relevant | Inactivation |
| *Enterococcus hirae* | Rare diarrhoea und septicaemia causing exciter [30][31]  commensal flora in birds [32] [33] | Sometimes clinically relevant | Inactivation |
| *Lactobacillus buchneri* | Fodder production [34] [35], bacteriocin former against Gram-positive e.g. enterocci [36], not against pediococci | Economically relevant  **fermentation MO** | Strong dynamics |
| *Lactobacillus harbinensis* | Food production, contamination in beer production [37] | Economically relevant  **fermentation MO** | Strong dynamics |
| *Lactobacillus paracasei* | Broad band bacteriocin former [38], discussed as (a)virulent ([39]) [40], [41], [42] | Not clinically relevant  **fermentation MO** | Strong dynamics |
| *Lactobacillus plantarum* | Food production [43], [44], Broad band bacteriocin former [45],[46] [47], therapeutic agent [48] | Not clinically relevant  economical relevant | Inactivation |
| *Lactobacillus salivarius* | Probiotic [49] [50], bacteriocin former [51] | No clinically relevant  economically relevant | Inactivation |
| *Pediococcus acidilactici* | Food production, bacteriocin former [52] [53], [54], potential virulent for immunocompromised [55], [41], [56] | Not clinically relevant  **fermentation MO** | Strong dynamics possible interaction with *L. plantarum* |
| *Staphylococcus fleurettii* | Milk associated MO [57], genus contains pathogens [58] | unknown, non pathogen, possible model organism | Inactivation |
| *Streptococcus lutetiensis* | Non pathogen, closely related to S. Bovis [59] , [60], genus contains pathogens [61] | unknown, non pathogen, possible model organism | Inactivation |

# Evaluation of species of matrix M

List of Gram-negative microorganisms of matrix **S**, their relevance and status during or after fermentation green: positive effect blue: neutral effect red: negative Effect

| **Species** | **Evaluation** | **Significance** | **During / After Fermentation** |
| --- | --- | --- | --- |
| *Acinetobacter* spp. | Nosocomial infectiv agent [62] | Increasingly clinically relevant [63] | Inactivation |
| *Acinetobacter junii* | Potential pathogen [64] | Sometimes clinically relevant | Inactivation |
| *Acinetobacter parvus* | Potential pathogen [65] | Sometimes clinically relevant | Inactivation |
| *Aeromonas caviae* | Sporadic toxinforming [66] | Low clinical relevance | Inactivation |
| *Aeromonas salmonicida* | Pathogen for fishes [67], [68] | Highly economically relevant | Inactivation |
| *Aeromonas veronii* | Pathogen [69] | Clinically relevant | Inactivation |
| *Citrobacter braakii* | Ocassional pathogen [70], pollution degrader [71] | Potentially economically and clinically relevant | Inactivation |
| *Citrobacter freundii* | Causes meningitis in newborn [72] | Highly clinically relevant for newborn | Inactivation |
| *Citrobacter gillennii* | Clinical isolates | Unknown clinical relevance [73] | Inactivation |
| *Citrobacter testosteroni* | Commensal flora | Sometimes clinically relevant [74] | Inactivation |
| *Enterobacter cloacae* | Nosocomial infectiv agent [75], [76] | Clinically relevant | Inactivation |
| *Enterobacter ludwigii* | Nosocomial infectiv agent [77] | Clinically relevant | Inactivation |
| *Escherichia coli* | Intestinal flora, pathogen [78], [2], [79] | Highly clinically relevant | Inactivation |
| *Klebsiella oxytoca* | sporadic intestinal flora, (nosocomial) pathogen [80], [81], [82], [83], [84] | Increasing clinical relevance | Inactivation |
| *Klebsiella pneumoniae* | Pathogen [81] | Highly clinically relevant | Inactivation |
| *Ochrobactrum anthropi* | Pathogen [85],[86], [87] | Clinically relevant | Inactivation |
| *Pandoraea sputorum* | Pathogen [88], [89] especialy relevant in cycstic fibrosis cases [90] | Clinically relevant | Inactivation |
| *Pseudomonas alcaligenes* | Soil MO [91], [92] clinical case [93] | Potentially clinically relevant | Inactivation |
| *Pseudomonas chlororaphis* | Non pathogen MO [94] , antagonist of phyto pathogen fungi [95] | No clinical relevance | Inactivation |
| *Pseudmonas corrugata* | Phyto pathogen [96], [97] | Economically relevant | Inactivation |
| *Pseudomonas monteilii* | Soil MO symbiotic mycorrhiza [98],  discussed as virulent [99] | Potentially economical relevant | Inactivation |
| *Pseudomonas nitroreducens* | Environment MO [100] pollution degrader [101] | Potentially economically relevant | Inactivation |
| *Pseudomonas stutzeri* | Environment MO [102], [103] low nosocomial potential [104], [105], [106] | Clinically relevant possible model organism for *Burkholderia* [107] | Inactivation |
| *Pseudomonas veronii* | Food spoiler [99], Xenobiotic degrader in sewage sludge [108][109] | Not clinically relevant  potentially economically relevant | Inactivation |
| *Serratia fonticola* | Pyogenic pathogen [110], [111] environmental MO [112] | Clinically relevant | Inactivation |
| *Serratia liquefaciens* | Environment MO, vertebrate associated [113] rhizosphere MO [114], psychrophil contaminant [115], [116], [117], [118] | Low clinical relevance | Inactivation |
| *Serratia marcescens* | Nosocomial pathogen [113] [119] , contaminant [120], [121], blood wonder [122], [123] | Clinically relevant | Inactivation |
| *Yersinia enterocolitica* | psychrophilic diarrhoea causing agent [124], many non pathogen biovars [124] | Sometimes Clinically relevant (found biovar group 1A [125]) | Inactivation |

List of Gram-positve microorganisms of matrix M, their relevance and status during or after fermentation. Green: positive effect blue: neutral effect red: negative effect

| **Species** | **Evaluation** | **Significance** | **During / After Fermentation** |
| --- | --- | --- | --- |
| *Bifidobacterium bifidum* | Intestinal MO, bacteriocide former [126], therapeutic [127] | No clinical relevance | Inactivation |
| *Bifidobacterium themophilum* | Intestinal MO, therapeutic [126] | No clinical relevance  **fermentation MO** | Strong dynamics |
| *Candida glabrata* | Commensal flora, [128] | Increasing clinical relevance [128] [129] | Inactivation |
| *Saccharomyces cerevisiae* | Brewery / bakery yeast [130] | Economically relevant, controversial clinical relevance [131] [132], [133], [134] | Inactivation |
| *Enterococcus avium* | Intestinal MO [135], rare bacteraemia causisng agent [136], [137] | Seldom clinicaly relevant | Inactivation |
| *Enterococcus aquimarinus* | Maritime MO [138], food production [139] | Economically relevant, Not clinically relevant | Inactivation |
| *E. casseliflavus* | Food production [140], virulent for immunocompromised patients [141] | Economically relevant, low clinical relevance | Inactivation |
| *Enterococcus faecalis* | Commensal and pathogen, cytolysin forming [142], [143] | High clinical relevance | Inactivation |
| *Enterococcus faecium* | Commensal and pathogen, less virulent than *E. faecalis* [26], [144] bacteriocide- and broad band bacteriocin former [28], [29] | High clinical relevance,  suspected pioneer flora in fermentation  **fermentation MO** | Strong dynamics and inactivation |
| *E. haemoperoxidus* | Waterborne MO | No clinical relevance | Inaktivierung |
| *Enterococcus hirae* | Uncommon diarrhoea and septicaemia causing agent [30], [145] Commensal MO in birds [146], [33] | seldom clinically relevant | Inaktivierung |
| *Enterococcus thailandicus* | Food production [147] | No clinical relevance | Inaktivierung |
| *Lactobacillus amylovorus* | Starch metabolizing, heterofermentative, sourdough MO [17], bacteriocide- and bacteriocin former [148], | No clinical relevance Economically relevant **fermentations-MO** | dynamics  possible interaction with *L.* *reuteri* (254) |
| *Lactobacillus brevis* | Food production [149], heterofermentative [150],  bacteriocide- and broad band bacteriocin former [151], [152], therapeutic [153] | No clinical relevance Economically relevant **fermentation MO** | dynamics |
| *Lactobacillus casei* | Avirulent [39], therapeutic [154], often used „functional food“ | No clinical relevance Highly economically relevant [155], potential fermentation MO | dynamics |
| *Lactobacillus crispatus* | broad band bacteriocin former [156], [157] | No clinical relevance High economical relevant **fermentation MO** | dynamics  possible interaction with *L. amylovorus and Pediococcus* sp. (230, 449) |
| *Lactobacillus delbrueckii* | narrow band bacteriocin former [157], [159] food production [160], controversial (a)virulent ([39]) [161] | No clinical relevance, highly economically relevant | dynamics |
| *Lactobacillus fermentum* | broad band bacteriocin former [162], food production [160], controversial (a)virulent ([39]) [161] | No clinical relevance, economically relevant, **fermentation MO** | Strong dynamics |
| *Lactobacillus gasseri* | bacteriocin former, antagonize pathogen MO, probiotic [39], [162], [164], [165], [157] sporicidal [166] | No clinical relevance, economically relevant  potential fermentations-MO | dynamics, possible interaction with *L. amylovorus a*nd *P. pentosaceus* (508) |
| *Lactobacillus kitasatonis* | broad band bacteriocin former [167] | No clinical relevance, economically relevant, **fermentation MO** | Strong dynamics |
| *Lactococcus lactis* | broad band bacteriocin former, sporicidal [168], [169], food production [170] | No clinical relevance, economically relevant, | Inactivation |
| *Lactobacillus mucosae* | First known mucosa adhesive *Lactobacillus*, antagonizes pathogen MO, broad band bacteriocin former [171], [172], [173] | No clinical relevance **fermentation MO** | Strong dynamics |
| *Lactobacillus oris* | Heterofermentative bacteriocin former [174], [175], [176], [177] | No clinical relevance potential fermentation MO | Dynamics, possible interaction with *L. crispatus* |
| *Lactobacillus parabuchneri* | Heterofermentative [178], food production [179] | No clinical relevance [178], economically relevant, potential climax population | Dynamics |
| *Lactobacillus paracasei* | broad band bacteriocin former [39] controversial (a)virulent ([39]) [40], [41], [42] | No clinical relevance **fermentation MO** | Strong dynamics |
| *Lactobacillus plantarum* | Food production [44],  broad band bacteriocin former [45], [46], [47], therapeutic [48] | No clinical relevance  **fermentation MO** | Inactivation |
| *Lactobacillus reuteri* | broad band bacteriocin former [180], [181] | No clinical relevance  **fermentation MO** | Dynamics, possible interaction with *L. amylovorans* |
| *Lactobacillus rhamnosus* | Positive influence on immune system [182] controversial (a)virulent ([39]) [40], [161], [183], [184], therapeutic [185] broad band bacteriocin former [184] | probably clinically relevant  potential fermentation MO | Strong dynamics |
| *Lactobacillus zeae* | Food Production [186] | No clinical relevance, potential climax population | Dynamics |
| *Micrococcus luteus* | Potential nosocomial infectiv agent [187] | increasing clinical relevance, probably  contamination in this case | Inactivation |
| *Pediococcus acidilactici* | Food production bacteriocin former [188], [53], controversial virulent [55], [56] | No clinical relevance,  **fermentation MO** | Strong dynamics, possible interaction with *L. plantarum* |
| *Pediococcus pentosaceus* | Sporocidal bacteriocin former [189], [190] potential virulent for immunocoimpromised patients [191], [192] | No clinical relevance,  **fermentation MO** | Strong dynamics, possible interaction with *L. reuteri,L. amylovorans* |
| *Staphylococcus epidermidis* | Skin MO, pyogenic exciter [193], [194] | clinically relevant | Inactivation |
| *Staphylococcus haemolyticus* | Skin MO, nosocomial infectiv agent [31], [195], [196], [197] | clinical relevance, probably contamination in this case | Inactivation |
| *Staphylococcus hominis* | Skin MO [198], pathogene subspecies [199] | Sometime clinically relevant | Inactivation |

List of Gram-positve spore forming microorganisms of matrix M, their relevance and status during or after fermentation. Green: positive effect blue: neutral effect red: negative effect

| **Species** | **Evaluation** | **Significance** | **During / After Fermentation** |
| --- | --- | --- | --- |
| *Bacillus arsenicus* | Environment MO [200]. | No clinical relevance | Survival in spore |
| *Bacillus cereus* | Food spoiler [8] potential model organism for *B. anthracis* [11] | clinical relevance | Survival in spore |
| *Bacillus flexus* | Commensal flora [201] | No clinical relevance | Survival in spore |
| *B. licheniformis* | Environment MO, rare food spoiler [9] | Usually No clinical relevance [10] | Survival in spore |
| *Bacillus megaterium* | Environment MO, rare food spoiler [9] | Usually No clinical relevance [202], [203] | Survival in spore |
| *Bacillus muralis* | Environment MO [204] | No clinical relevance | Survival in spore |
| *Bacillus mycoides* | Non pathogen leave flora, *B. cereus – g*roup [205] | Antagonize phyto pathogens [206] | Survival in spore |
| *Bacillus pseudomycoides* | Environment MO, *B. cereus – group* [205] | No clinical relevance | Survival in spore |
| *B. pumilus* | Soil MO, supports plant grow | Antagonize phyto pathogens [13] | Survival in spore |
| *B. simplex* | Soil MO, rare food spoiler [9] | Usualy no clinical relevance | Survival in spore |
| *B. subtilis* | Antifungal [207] | agricultural relevance [208] | Survival in spore |
| *Bacillus thuringiensis* | Antifungal insecticide [209] | agricultural relevance | Survival in spore |
| *Bacillus weihenstepahnensis* | Toxin former, potential food spoiler *B. cereus – group* [210], [208], [212] | Usualy no clinical relevance | Survival in spore |
| *Clostridium baratii* | Potential botulism – causing agent [213], [214], [215] | Potential clinical relevance | Inactivation (douptful because of spore forming capacity) |
| *Clostridium bifermentans* | Controversial virulent [216], [217] | Potential clinical relevance | Dynamics |
| *Clostridium butyricum* | Potential botulism – causing agent [218] | Potential clinical relevance | Inactivation (douptful because of spore forming capacity) |
| *Clostridium bolteae* | Controversial autism causing agent [219], [220], [221] | Potential clinical relevance | Inactivation (douptful because of spore forming capacity) |
| *Clostridium celerecrescens* | Cellulose degrader [222], extremly rare clinical [223] | No clinical relevance | Dynamics |
| *Clostridium clostridioforme* | Earlier discussed as avirulent [216]*,*  Controversial autism causing agent [219], [220], [221] | Potential clinical relevance | Strong dynamics |
| *Clostridium cochlearium* | Environment MO | Biochemical relevance, No clinical relevance [224], [225] | Slight reduction |
| *Clostridium colicanis* | Carnivores associated MO [226], uncommon bacteraemia causing agent | Potential clinical relevance [140] | Slight reduction |
| *Clostridium disporicum* | Environment MO [227] extremly rare clinical [228] | Potential clinical relevance | Survival in spore |
| *Clostridium paraputrificum* | Environment MO, chitinase activity [229] | Usually no clinical relevance | Slight reduction |
| *Clostridium perfringens* | Intestinal and Soil MO, pathogen [230], [2], [231], [232] | High clinically relevance | Slight reduction |
| *Clostridium sordellii* | Vaginal MO [233], pathogen [234] | High clinically relevance | Survival in spore |
| *Clostridium sporogenes* | Soil MO, rare pathogen [235], [236] | Rare clinically relevance | Survival in spore |
| *Lysinibacillus fusiformis* | Soil MO [237], fungicide former [238], biofilm inhibitor [239], potential reservoir for antibiotic resisance [240] | Potential tenazitätssenkend [241] | Survival in spore |
| *Paenibacillus amylolyticus* | Environment MO | No clinically relevance | Pot. Überdauerung in Spore |
| *Paenibacillus glucanolyticus* | Soil MO [242], potential pathogen for insects [243], extremly rare human pathogenic [244] | Rare clinically relevance | Survival in spore |
| *Paenibacillus lactis* | Food spoiler [245] | No clinically relevance | Survival in spore |
| *Paenibacillus lautus* | Soil and intestinal MO [246] | No clinically relevance | Potential survival in spore |

1. Capdevila JA, Bisbe V, Gasser I, Zuazu J, Olivé T, et al. (1998) Enterobacter amnigenus. An unusual human pathogen. Enfermedades infecciosas y microbiología clínica 16: 364.

2. Quinn PJ, Markey BK, Leonard FC, FitzPatrick ES, Fanning S, et al. (2011) Veterinary microbiology and microbial disease. Second Edition. Chichester and West Sussex and UK: Wiley-Blackwell.

3. Cruz AT, Cazacu AC, Allen CH (2007) Pantoea agglomerans, a Plant Pathogen Causing Human Disease. Journal of Clinical Microbiology 45: 1989–1992. doi:10.1128/JCM.00632-07.

4. Jackson T., Wang H, Nugent MJ, Griffin CT, Burnell AM, et al. (1995) Isolation of insect pathogenic bacteria, Providencia rettgeri, from Heterorhabditis spp. Journal of Applied Microbiology 78: 237–244. doi:10.1111/j.1365-2672.1995.tb05022.x.

5. Yoh M (2005) Importance of Providencia species as a major cause of travellers’ diarrhoea. Journal of Medical Microbiology 54: 1077–1082. doi:10.1099/jmm.0.45846-0.

6. Hildebrand DC, Palleroni NJ, Hendson M, Toth J, Johnson JL (1994) Pseudomonas flavescens sp. nov., Isolated from Walnut Blight Cankers. International Journal of Systematic Bacteriology 44: 410–415. doi:10.1099/00207713-44-3-410.

7. Palleroni NJ (1992) Human- and Animal-Pathogenic Pseudomonads. In: Balows A, Trüpfer HG, Dworkin M, Harder W, Schleifer K-H, editors. The prokaryotes. New York and NY and Berlin and Heidelberg: Springer. pp. 3087–3103.

8. Farrar WE, Reboli AC (1992) The Genus Bacillus - Medical. In: Balows A, Trüpfer HG, Dworkin M, Harder W, Schleifer K-H, editors. The prokaryotes. New York and NY and Berlin and Heidelberg: Springer. pp. 1746–1768.

9. Taylor JM., Sutherland AD, Aidoo KE, Logan NA (2005) Heat-stable toxin production by strains of Bacillus cereus , Bacillus firmus , Bacillus megaterium , Bacillus simplex and Bacillus licheniformis. FEMS Microbiology Letters 242: 313–317. doi:10.1016/j.femsle.2004.11.022.

10. Hanna P (1998) Anthrax pathogenesis and host response. In: Vogt PK, Mahan MJ, editors. Bacterial Infection: Close Encounters at the Host Pathogen Interface. Current Topics in Microbiology and Immunology. Berlin and Heidelberg: Springer Berlin Heidelberg, Vol. 225. pp. 13–35.

11. Helgason E, Okstad OA, Caugant DA, Johansen HA, Fouet A, et al. (2000) Bacillus anthracis, Bacillus cereus, and Bacillus thuringiensis—One Species on the Basis of Genetic Evidence. Applied and Environmental Microbiology 66: 2627–2630. doi:10.1128/AEM.66.6.2627-2630.2000.

12. Veith B, Herzberg C, Steckel S, Feesche J, rg, et al. (2004) The Complete Genome Sequence of Bacillus licheniformis DSM13, an Organism with Great Industrial Potential. Journal of Molecular Microbiology and Biotechnology 7: 204–211. doi:10.1159/000079829.

13. Manero GF., Acero N, Lucas J., Probanza A (1996) The infuence of native rhizobacteria on European alder (Alnus glutinosa (L.) Gaertn.) growth. Plant and Soil 182: 67–74.

14. Logan NA (2002) Polyphasic identification of Bacillus and Brevibacillus strains from clinical, dairy and industrial specimens and proposal of Brevibacillus invocatus sp. nov. International Journal of Systematic and Evolutionary Microbiology 52: 953–966. doi:10.1099/ijs.0.02081-0.

15. Sanders M., Morelli L, Tompkins T. (2003) Sporeformers as Human Probiotics: Bacillus, Sporolactobacillus, and Brevibacillus. Comprehensive Reviews in Food Science and Food Safety 2: 101–110. doi:10.1111/j.1541-4337.2003.tb00017.x.

16. Laubach CA, Rice JL (1916) Spore-Bearing Bacteria in Soil: AEROBIC SPORE-BEARING NON-PATHOGENIC BACTERIA. Journal of Applied Bacteriology 1: 513–518.

17. Nakamura LK (1993) DNA Relatedness of Bacillus brevis Migula 1900 Strains and Proposal of Bacillus agri sp. nov., nom. rev., and Bacillus centrosporus sp. nov., nom. rev. International Journal of Systematic Bacteriology 43: 20–25. doi:10.1099/00207713-43-1-20.

18. SHIDA O, TAKAGI H, KADOWAKI K, KOMAGATA K (1996) Proposal for Two New Genera, Brevibacillus gen. nov. and Aneurinibacillus gen. nov. International Journal of Systematic Bacteriology 46: 939–946. doi:10.1099/00207713-46-4-939.

19. Kongpol A, Pongtharangkul T, Kato J, Honda K, Ohtake H, et al. (2009) Characterization of an organic-solvent-tolerant Brevibacillus agri strain 13 able to stabilize solvent/water emulsion. FEMS Microbiology Letters 297: 225–233. doi:10.1111/j.1574-6968.2009.01684.x.

20. Adam D, editor (2004) Die Infektiologie: Mit 444 Tabellen. Berlin and Heidelberg [u.a.]: Springer.

21. Abe F, Ishibashi N, Shimamura S (1995) Effect of Administration of Bifidobacteria and Lactic Acid Bacteria to Newborn Calves and Piglets. Journal of Dairy Science 78: 2838–2846. doi:10.3168/jds.S0022-0302(95)76914-4.

22. Meng Q, Kerley MS, Russel TJ, Allee GL (1998) Lectin-like activity of Escherichia coli K88, Salmonella choleraesuis, and Bifidobacteria pseudolongum of porcine gastrointestinal origin. Journal of animal science 76: 551–556.

23. Monnet C, Correia K, Sarthou A-S, Irlinger F (2006) Quantitative detection of Corynebacterium casei in cheese by real-time PCR. Applied and environmental microbiology 72: 6972–6979.

24. Graevenitz A von, Krech T (1992) The Genus Corynebacterium - Medical. In: Balows A, Trüpfer HG, Dworkin M, Harder W, Schleifer K-H, editors. The prokaryotes. New York and NY and Berlin and Heidelberg: Springer. pp. 1172–1187.

25. Van Schaik W, Top J, Riley DR, Boekhorst J, Vrijenhoek JEP, et al. (2010) Pyrosequencing-based comparative genome analysis of the nosocomial pathogen Enterococcus faecium and identification of a large transferable pathogenicity island. BMC Genomics 11: 239. doi:10.1186/1471-2164-11-239.

26. Huycke MM, Sahm D., Gilmore M. (1998) Multiple-drug resistant enterococci: the nature of the problem and an agenda for the future. Emerging Infectious Diseases 4: 239–249.

27. Vandenbergh PA (1993) Lactic acid bacteria, their metabolic products and interference with microbial growth. FEMS Microbiology Reviews.

28. Cintas LM, Casaus P, Havarstein LS, Hernández PE, Nes IF (1997) Biochemical and genetic characterization of enterocin P, a novel sec-dependent bacteriocin from Enterococcus faecium P13 with a broad antimicrobial spectrum. Applied and Environmental Microbiology 63: 4321–4330.

29. Leblanc DJ (2006) Enterococcus. In: Dworkin M, Falkow S, Rosenberg E, Schleifer K-H, Stackebrandt E, editors. The Prokaryotes. New York and NY: Springer US. pp. 175–204.

30. Etheridge ME, Yolken RH, Vonderfecht S. L. (1988) Enterococcus hirae implicated as a cause of diarrhea in suckling rats. Journal of Clinical Microbiology 26: 1741–1744.

31. Froggatt JW, Johnston JL, Galetto DW, Archer GL (1989) Antimicrobial resistance in nosocomial isolates of Staphylococcus haemolyticus. Antimicrobial Agents and Chemotherapy 33: 460–466. doi:10.1128/AAC.33.4.460.

32. Svec P, Devriese LA, Sedlácek I, Baele M, Vancanneyt M, et al. (2001) Enterococcus haemoperoxidus sp. nov. and Enterococcus moraviensis sp. nov., isolated from water. International Journal of Systematic and Evolutionary Microbiology 51: 1567–1574.

33. Farrow JAE, Collins MD (1985) Enterococcus hirae, a New Species That Includes Amino Acid Assay Strain NCDO 1258 and Strains Causing Growth Depression in Young Chickens. International Journal of Systematic Bacteriology 35: 73–75. doi:10.1099/00207713-35-1-73.

34. Driehuis F, Oude Elferink SJWH, van Wikselaar PG (2001) Fermentation characteristics and aerobic stability of grass silage inoculated with Lactobacillus buchneri, with or without homofermentative lactic acid bacteria. Grass and Forage Science 56: 330–343. doi:10.1046/j.1365-2494.2001.00282.x.

35. Ranjit N., Kung L (2000) The Effect of Lactobacillus buchneri, Lactobacillus plantarum, or a Chemical Preservative on the Fermentation and Aerobic Stability of Corn Silage. Journal of Dairy Science 83: 526–535. doi:10.3168/jds.S0022-0302(00)74912-5.

36. Yıldırım Z, Yıldırım M (2002) Factors affecting the adsorption of buchnericin LB, a bacteriocin produced by< i> Lactocobacillus buchneri</i>. Microbiological research 157: 103–107.

37. Miyamoto M, Seto Y, Hai Hao D, Teshima T, Bo Sun Y, et al. (2005) Lactobacillus harbinensis sp. nov., consisted of strains isolated from traditional fermented vegetables `Suan cai’ in Harbin, Northeastern China and Lactobacillus perolens DSM 12745. Systematic and Applied Microbiology 28: 688–694. doi:10.1016/j.syapm.2005.04.001.

38. Lozo J, Vukasinovic M, Strahinic I, Topisirovic A (2004) Characterization and Antimicrobial Activity of Bacteriocin 217 Produced by Natural Isolate Lactobacillus paracasei subsp. paracasei BGBUK2-16. Journal of Food Protection: 2727–2734.

39. Hammes WP, Hertel C (2006) The Genera Lactobacillus and Carnobacterium. In: Dworkin M, Falkow S, Rosenberg E, Schleifer K-H, Stackebrandt E, editors. The Prokaryotes. New York and NY: Springer US. pp. 320–403.

40. Cannon JP, Lee TA, Bolanos JT, Danziger LH (2005) Pathogenic relevance of Lactobacillus: a retrospective review of over 200 cases. European Journal of Clinical Microbiology & Infectious Diseases 24: 31–40. doi:10.1007/s10096-004-1253-y.

41. Mastro TD, Spika JS, Lozano P, Appel J, Facklam RR (1990) Vancomycin-Resistant Pediococcus acidilactici: Nine Cases of Bacteremia. Journal of Infectious Diseases 161: 956–960. doi:10.1093/infdis/161.5.956.

42. Saxelin M, Chuang N-H, Chassy B, Rautelin H, Makela PH, et al. (1996) Lactobacilli and Bacteremia in Southern Finland, 1989-1992. Clinical Infectious Diseases 22: 564–566. doi:10.1093/clinids/22.3.564.

43. Eghball B, Power JF, Gilley JE, Doran JW (1997) Nutrient, carbon, and mass loss during composting of beef cattle feedlot manure. Journal of environmental quality 26: 189–193.

44. Vries MC de, Vaughan EE, Kleerebezem M, Vos WM de (2006) Lactobacillus plantarum—survival, functional and potential probiotic properties in the human intestinal tract. International Dairy Journal 16: 1018–1028. doi:10.1016/j.idairyj.2005.09.003.

45. Jiménez-Díaz R, Rios-Sanchez RM, Desmazeaud M, Ruiz-Barba JL, Piard JC (1993) Plantaricins S and T, two new bacteriocins produced by Lactobacillus plantarum LPCO10 isolated from a green olive fermentation. Applied and Environmental Microbiology 59: 1416–1424.

46. Todorov S., Dicks LM. (2005) Lactobacillus plantarum isolated from molasses produces bacteriocins active against Gram-negative bacteria. Enzyme and Microbial Technology 36: 318–326. doi:10.1016/j.enzmictec.2004.09.009.

47. van Reenen, Dicks, Chikindas (1998) Isolation, purification and partial characterization of plantaricin 423, a bacteriocin produced by Lactobacillus plantarum. Journal of Applied Microbiology 84: 1131–1137. doi:10.1046/j.1365-2672.1998.00451.x.

48. Niedzielin K, Kordecki H, Birkenfeld B ena (2001) A controlled, double-blind, randomized study on the efficacy of Lactobacillus plantarum 299V in patients with irritable bowel syndrome. European Journal of Gastroenterology & Hepatology 13: 1143–1147. doi:10.1097/00042737-200110000-00004.

49. Avía Y, Suzuki N, Kabir AMA, Takagi A, Koga Y (1998) Lactic acidmediated suppression of Helicobacter pylori by the oral administration of Lactobacillus salivarius as a probiotic in a gnotobiotic murine model. Am J Gastroenterol 93: 2097–2101.

50. Hammes W., Weiss N, Holzapfel W (1992) The Genera Lactobacillus and Carnobacterium. In: Balows A, Trüpfer HG, Dworkin M, Harder W, Schleifer K-H, editors. The prokaryotes. New York and NY and Berlin and Heidelberg: Springer. pp. 1535–1595.

51. Corr SC, Li Y, Riedel CU, O’Toole PW, Hill C, et al. (2007) Bacteriocin production as a mechanism for the antiinfective activity of Lactobacillus salivarius UCC118. Proceedings of the National Academy of Sciences 104: 7617–7621.

52. Bhunia A., Johnson M., Ray B, Kalchayanand N (1991) Mode of action of pediocin AcH from Pediococcus acidilactici H on sensitive bacterial strains. Journal of Applied Bacteriology 70: 25–33. doi:10.1111/j.1365-2672.1991.tb03782.x.

53. Cintas LM, Rodriguez JM, Fernandez MF, Sletten K, Nes IF, et al. (1995) Isolation and characterization of pediocin L50, a new bacteriocin from Pediococcus acidilactici with a broad inhibitory spectrum. Applied and Environmental Microbiology 61: 2643–2648.

54. Ray B, Daeschel M. (1994) Bacteriocins of Starter Culture Bacteria. In: Dillon V., Board R., editors. Natural antimicrobial systems and foord preservation. Wallingford UK: Biddles Ltd, Guildford.

55. Golledge CL, Stingemore N, Aravena M, Joske D (1990) Septicemia caused by vancomycin-resistant Pediococcus acidilactici. Journal of Clinical Microbiology 28: 1678–1679.

56. Sarma PS, Mohanty S (1998) Pediococcus acidilactici pneumonitis and bacteremia in a pregnant woman. Journal of Clinical Microbiology 36: 2392–2393.

57. Vernozy-Rozand C, Mazuy C, Meugnier H, Bes M, Lasne Y, et al. (2000) Staphylococcus fleurettii sp. nov., isolated from goat’s milk cheeses. International journal of systematic and evolutionary microbiology 50: 1521–1527.

58. Kloos EW, Schleifer K-H, Götz F (1992) The Genus Staphylococcus. In: Balows A, Trüpfer HG, Dworkin M, Harder W, Schleifer K-H, editors. The prokaryotes. New York and NY and Berlin and Heidelberg: Springer. pp. 1369–1420.

59. Poyart C (2002) Taxonomic dissection of the Streptococcus bovis group by analysis of manganese-dependent superoxide dismutase gene (sodA) sequences: reclassification of “Streptococcus infantarius subsp. coli” as Streptococcus lutetiensis sp. nov. and of Streptococcus bovis biotype II.2 as Streptococcus pasteurianus sp. nov. International Journal of Systematic and Evolutionary Microbiology 52: 1247–1255. doi:10.1099/ijs.0.02044-0.

60. Romero B, Morosini M-I, Loza E, Rodriguez-Banos M, Navas E, et al. (2011) Reidentification of Streptococcus bovis Isolates Causing Bacteremia According to the New Taxonomy Criteria: Still an Issue? Journal of Clinical Microbiology 49: 3228–3233. doi:10.1128/JCM.00524-11.

61. Ruoff KL (1992) The Genus Streptococcus - Medical. In: Balows A, Trüpfer HG, Dworkin M, Harder W, Schleifer K-H, editors. The prokaryotes. New York and NY and Berlin and Heidelberg: Springer. pp. 1450–1464.

62. Joly-Guillou M-L (2005) Clinical impact and pathogenicity of Acinetobacter. Clinical Microbiology and Infection 11: 868–873. doi:10.1111/j.1469-0691.2005.01227.x.

63. Rello J (1999) <italic>Acinetobacter baumannii</italic> Infections in the ICU <subtitle>Customization is the Key</subtitle>. CHEST Journal 115: 1226. doi:10.1378/chest.115.5.1226.

64. Bernards A., Beaufort A. de, Dijkshoorn L, van Boven CP. (1997) Outbreak of septicaemia in neonates caused by Acinetobacter junii investigated by amplified ribosomal DNA restriction analysis (ARDRA) and four typing methods. Journal of Hospital Infection 35: 129–140. doi:10.1016/S0195-6701(97)90101-8.

65. Nemec A (2003) Acinetobacter parvus sp. nov., a small-colony-forming species isolated from human clinical specimens. International Journal of Systematic and Evolutionary Microbiology 53: 1563–1567. doi:10.1099/ijs.0.02631-0.

66. Callister SM, Agger WA (1987) Enumeration and characterization of Aeromonas hydrophila and Aeromonas caviae isolated from grocery store produce. 53.

67. Farmer III J., Arduino M., Hickman-Brenner F. (1992) The Genera Aeromonas und Plesiomonas. In: Balows A, Trüpfer HG, Dworkin M, Harder W, Schleifer K-H, editors. The prokaryotes. New York and NY and Berlin and Heidelberg: Springer. pp. 3012–3045.

68. SCOTT M (1968) The Pathogenicity of Aeromonas salmonicida (Griffin) in Sea and Brackish Waters. Journal of General Microbiology 50: 321–327. doi:10.1099/00221287-50-2-321.

69. Rahman M, Colque-Navarro P, Kuhn I, Huys G, Swings J, et al. (2002) Identification and Characterization of Pathogenic Aeromonas veronii Biovar Sobria Associated with Epizootic Ulcerative Syndrome in Fish in Bangladesh. Applied and Environmental Microbiology 68: 650–655. doi:10.1128/AEM.68.2.650-655.2002.

70. Brenner DJ, GRIMONT PAD, Steigerwalt AG, FANNING GR, AGERON E, et al. (1993) Classification of Citrobacteria by DNA Hybridization: Designation of Citrobacter farmeri sp. nov., Citrobacter youngae sp. nov., Citrobacter braakii sp. nov., Citrobacter werkmanii sp. nov., Citrobacter sedlakii sp. nov., and Three Unnamed Citrobacter Genomospecies. International Journal of Systematic Bacteriology 43: 645–658. doi:10.1099/00207713-43-4-645.

71. Dhouib A, Hamad N, Hassaı̈ri I, Sayadi S (2003) Degradation of anionic surfactants by Citrobacter braakii. Process Biochemistry 38: 1245–1250. doi:10.1016/S0032-9592(02)00322-9.

72. Badger JL, Stins MF, Kim KS (1999) Citrobacter freundii Invades and Replicates in Human Brain Microvascular Endothelial Cells. Infection and Immunity 67: 4208–4215.

73. Brenner DJ, O’Hara CM, Grimont PA, Janda JM, Falsen E, et al. (1999) Biochemical Identification of Citrobacter Species Defined by DNA Hybridization and Description of Citrobacter gillenii sp. nov. (FormerlyCitrobacter Genomospecies 10) and Citrobacter murliniae sp. nov. (Formerly CitrobacterGenomospecies 11). Journal of Clinical Microbiology 37: 2619–2624.

74. Abraham JM, Simon GL (2007) Comamonas testosteroni Bacteremia. Infectious Diseases in Clinical Practice 15: 272–273. doi:10.1097/IPC.0b013e31802ce475.

75. Harbarth S, Sudre P, Dharan S, Cadenas M, Pittet D (1999) Outbreak of Enterobacter cloacae Related to Understaffing, Overcrowding, and Poor Hygiene Practices •. Infection Control and Hospital Epidemiology 20: 598–603. doi:10.1086/501677.

76. Tzelepi E, Giakkoupi P, Sofianou D, Loukova V, Kemeroglou A, et al. (2000) Detection of Extended-Spectrum \textgreekb-Lactamases in Clinical Isolates of Enterobacter cloacae andEnterobacter aerogenes. Journal of Clinical Microbiology 38: 542–546.

77. Hoffmann H, Stindl S, Stumpf A, Mehlen A, Monget D, et al. (2005) Description of Enterobacter ludwigii sp. nov., a novel Enterobacter species of clinical relevance. Systematic and Applied Microbiology 28: 206–212. doi:10.1016/j.syapm.2004.12.009.

78. Naylor SW, Low JC, Besser TE, Mahajan A, Gunn GJ, et al. (2003) Lymphoid Follicle-Dense Mucosa at the Terminal Rectum Is the Principal Site of Colonization of Enterohemorrhagic Escherichia coli O157:H7 in the Bovine Host. Infection and Immunity 71: 1505–1512. doi:10.1128/IAI.71.3.1505-1512.2003.

79. KRUIS W, SCHUTZ E, FRIC P, FIXA B, JUDMAIER G, et al. (1997) Double-blind comparison of an oral Escherichia coli preparation and mesalazine in maintaining remission of ulcerative colitis. Alimentary Pharmacology and Therapeutics 11: 853–858. doi:10.1046/j.1365-2036.1997.00225.x.

80. Beaugerie L, Metz M, Barbut F, Bellaiche G, Petit J-C (2003) Klebsiella oxytoca as an agent of antibiotic-associated hemorrhagic colitis. Clinical Gastroenterology and Hepatology 1: 370–376.

81. Highsmith AK, Jarvis WR (1985) Klebsiella pneumoniae: Selected Virulence Factors That Contribute to Pathogenicity. Infection Control 6: 75–77.

82. Högenauer C, Langner C, Beubler E, Lippe IT, Schicho R, et al. (2006) Klebsiella oxytoca as a Causative Organism of Antibiotic-Associated Hemorrhagic Colitis. New England Journal of Medicine 355: 2418–2426. doi:10.1056/NEJMoa054765.

83. Lautenbach E, Patel JB, Bilker WB, Edelstein PH, Fishman NO (2001) Extended-Spectrum -Lactamase-Producing Escherichia coli and Klebsiella pneumoniae: Risk Factors for Infection and Impact of Resistance on Outcomes. Clinical Infectious Diseases 32: 1162–1171. doi:10.1086/319757.

84. Yong D, Toleman MA, Giske CG, Cho HS, Sundman K, et al. (2009) Characterization of a New Metallo- -Lactamase Gene, blaNDM-1, and a Novel Erythromycin Esterase Gene Carried on a Unique Genetic Structure in Klebsiella pneumoniae Sequence Type 14 from India. Antimicrobial Agents and Chemotherapy 53: 5046–5054. doi:10.1128/AAC.00774-09.

85. Alnor D, Frimodt-Meller N, Espersen F, Frederiksen W (1994) Infections with the Unusual Human Pathogens Agrobacterium Species and Ochrobactrum anthropi. Clinical Infectious Diseases 18: 914–920. doi:10.1093/clinids/18.6.914.

86. Cieslak TJ, Drabick CJ, Robb ML (1996) Pyogenic Infections Due to Ochrobactrum anthropi. Clinical Infectious Diseases 22: 845–847. doi:10.1093/clinids/22.5.845.

87. HOLMES B, POPOFF M, KIREDJIAN M, KERSTERS K (1988) Ochrobactrum anthropi gen. nov., sp. nov. from Human Clinical Specimens and Previously Known as Group Vd. International Journal of Systematic Bacteriology 38: 406–416. doi:10.1099/00207713-38-4-406.

88. Coenye T, Falsen E, Hoste B, Ohlen M, Goris J, et al. (2000) Description of Pandoraea gen. nov. with Pandoraea apista sp. nov., Pandoraea pulmonicola sp. nov., Pandoraea pnomenusa sp. nov., Pandoraea sputorum sp. nov. and Pandoraea norimbergensis comb. nov. International Journal of Systematic and Evolutionary Microbiology 50: 887–899. doi:10.1099/00207713-50-2-887.

89. Fernandez-Olmos A, Morosini MI, Lamas A, Garcia-Castillo M, Garcia-Garcia L, et al. (2012) Clinical and Microbiological Features of a Cystic Fibrosis Patient Chronically Colonized with Pandoraea sputorum Identified by Combining 16S rRNA Sequencing and Matrix-Assisted Laser Desorption Ionization-Time of Flight Mass Spectrometry. Journal of Clinical Microbiology 50: 1096–1098. doi:10.1128/JCM.05730-11.

90. Franke U (2008) Mukoviszidose: Cystische Fibrose. 1st ed. s.l: GRIN Verlag. Available: http://ebooks.ciando.com/book/index.cfm/bok_id/116681.

91. Akhtar MS, Siddiqui ZA (2008) Glomus intraradices, Pseudomonas alcaligenes, and Bacillus pumilus: effective agents for the control of root-rot disease complex of chickpea (Cicer arietinum L.). Journal of General Plant Pathology 74: 53–60. doi:10.1007/s10327-007-0062-4.

92. Stanier RY, Palleroni NJ, Doudoroff M (1966) The aerobic pseudomonads a taxonomic study. Journal of General Microbiology 43: 159–271.

93. Valenstein P, Bardy GH, Cox CC, Zwadyk P (1983) Pseudomonas alcaligenes endocarditis. American journal of clinical pathology 79: 245.

94. Gunther NW, Nunez A, Fett W, Solaiman DKY (2005) Production of Rhamnolipids by Pseudomonas chlororaphis, a Nonpathogenic Bacterium. Applied and Environmental Microbiology 71: 2288–2293. doi:10.1128/AEM.71.5.2288-2293.2005.

95. Tombolini R, van der Gaag DJ, Gerhardson B, Jansson JK (1999) Colonization Pattern of the Biocontrol StrainPseudomonas chlororaphis MA 342 on Barley Seeds Visualized by Using Green Fluorescent Protein. Applied and Environmental Microbiology 65: 3674–3680.

96. Catara V, Sutra L, Morineau A, Achouak W, Christen R, et al. (2002) Phenotypic and genomic evidence for the revision of Pseudomonas corrugata and proposal of Pseudomonas mediterranea sp. nov. International Journal of Systematic and Evolutionary Microbiology 52: 1749–1758.

97. SCARLETT CM, FLETCHER JT, ROBERTS P, LELLIOTT RA (1978) Tomato pith necrosis caused by Pseudomonas corrugata n. sp. Annals of Applied Biology 88: 105–114. doi:10.1111/j.1744-7348.1978.tb00684.x.

98. Duponnois R, Kisa M (2006) The possible role of trehalose in the mycorrhiza helper bacterium effect. Canadian Journal of Botany 84: 1005–1008. doi:10.1139/b06-053.

99. Elomari M, Coroler L, Verhille S, Izard D, Leclerc H (1997) Pseudomonas monteilii sp. nov., isolated from clinical specimens. International Journal of Systematic Bacteriology 47: 846–852.

100. IIZUKA H, Komagata K (1964) MICROBIOLOGICAL STUDIES ON PETROLEUM AND NATURAL GAS. The Journal of General and Applied Microbiology 10: 207–221. doi:10.2323/jgam.10.207.

101. Zhang H, Wan H, Song L, Jiang H, Wang H, et al. (2010) Development of an autofluorescent Pseudomonas nitroreducens with dehydrochlorinase activity for efficient mineralization of \textgreekg-hexachlorocyclohexane (\textgreekg-HCH). Journal of Biotechnology 146: 114–119. doi:10.1016/j.jbiotec.2010.01.020.

102. Palleroni NJ, DOUDOROFF M, Stanier RY, SOLaNES RE, MANDEL M (1970) Taxonomy of the Aerobic Pseudomonads: the Properties of the Pseudomonas stutzeri Group. Journal of General Microbiology 60: 215–231. doi:10.1099/00221287-60-2-215.

103. Rossello R, Garcia-Valdes E, Lalucat J, Ursing J (1991) Genotypic and Phenotypic Diversity of Pseudomonas stutzeri. Systematic and Applied Microbiology 14: 150–157. doi:10.1016/S0723-2020(11)80294-8.

104. Goetz A (1983) Pseudomonas stutzeri Bacteremia Associated With Hemodialysis. Archives of Internal Medicine 143: 1909. doi:10.1001/archinte.1983.00350100073018.

105. Keys TF, Melton LJ, Maker MD, Ilstrup DM (1983) A suspected hospital outbreak of pseudobacteremia due to Pseudomonas stutzeri. Journal of Infectious Diseases 147: 489–493.

106. Noble RC, Overman SB (1994) Pseudomonas stutzeri infection a review of hospital isolates and a review of the literature. Diagnostic Microbiology and Infectious Disease 19: 51–56. doi:10.1016/0732-8893(94)90051-5.

107. Yabuuchi E, Kosako Y, Oyaizu H, Yano I, Hotta H, et al. (1991) Proposal of Burkholderia gen. nov. and transfer of seven species of the genus Pseudomonas homology group II to the new genus, with the type species Burkholderia cepacia (Palleroni and Holmes 1981) comb. nov. Microbiology and immunology 36: 1251–1275.

108. Ajithkumar B, Ajithkumar VP, Iriye R (2003) Degradation of 4-amylphenol and 4-hexylphenol by a new activated sludge isolate of Pseudomonas veronii and proposal for a new subspecies status. Research in Microbiology 154: 17–23. doi:10.1016/S0923-2508(02)00009-8.

109. Nam I-H, Chang Y-S, Hong H-B, Lee Y-E (2003) A novel catabolic activity of Pseudomonas veronii in biotransformation of pentachlorophenol. Applied Microbiology and Biotechnology 62: 284–290. doi:10.1007/s00253-003-1255-1.

110. Bollet C, Gainnier M, Sainty JM, Orhesser P, Micco P de (1991) Serratia fonticola isolated from a leg abscess. Journal of Clinical Microbiology 29: 834.

111. Pfyffer GE (1992) Serratia fonticola as an infectious agent. European Journal of Clinical Microbiology & Infectious Diseases 11: 199–200. doi:10.1007/BF01967080.

112. Hopkins MJ, Macfarlane GT (2002) Changes in predominant bacterial populations in human faeces with age and with Clostridium difficile infection. Journal of Medical Microbiology 51: 448–454.

113. Grimont F, Grimont PAD (2006) The Genus Serratia. In: Dworkin M, Falkow S, Rosenberg E, Schleifer K-H, Stackebrandt E, editors. The Prokaryotes. Springer New York. pp. 219–244.

114. Kalbe C, Marten P, Berg G (1996) Strains of the genus Serratia as beneficial rhizobacteria of oilseed rape with antifungal properties. Microbiological Research 151: 433–439. doi:10.1016/S0944-5013(96)80014-0.

115. Boulton, Chapman, Walsh (1998) Fatal reaction to transfusion of red-cell concentrate contaminated with Serratia liquefaciens. Transfusion Medicine 8: 15–18. doi:10.1046/j.1365-3148.1998.00119.x.

116. Dainty RH, Edwards RA, Hibbard CM, Ramantanis SV (1986) Bacterial sources of putrescine and cadaverine in chill stored vacuum-packaged beef. Journal of Applied Bacteriology 61: 117–123. doi:10.1111/j.1365-2672.1986.tb04264.x.

117. Grohskopf LA, Roth VR, Feikin DR, Arduino MJ, Carson LA, et al. (2001) Serratia liquefaciens Bloodstream Infections from Contamination of Epoetin Alfa at a Hemodialysis Center. New England Journal of Medicine 344: 1491–1497. doi:10.1056/NEJM200105173442001.

118. Roth V., Arduino M., Nobiletti J, Holt S., Carson L., et al. (2000) Transfusion-related sepsis due to Serratia liquefaciens in the United States. Transfusion 40: 931–935. doi:10.1046/j.1537-2995.2000.40080931.x.

119. HEJAZI A, FALKINER FR (1997) Serratia marcescens. Journal of Medical Microbiology 46: 903–912. doi:10.1099/00222615-46-11-903.

120. Marrie TJ, Costerton JW (1981) Prolonged survival of Serratia marcescens in chlorhexidine. Applied and Environmental Microbiology 42: 1093–1102.

121. Szewzyk U, Szewzyk R, Stenström TA (1993) Growth and survival of Serratia marcescens under aerobic and anaerobic conditions in the presence of materials from blood bags. Journal of Clinical Microbiology 31: 1826–1830.

122. Köhler W (1992) Pest, Pestheilige, Blutwunder und andere Begebenheiten aus der Geschichte der Bakteriologie. Leopoldina 37: 211–238.

123. Yu VL (1979) Serratia marcescens. New England Journal of Medicine 300: 887–893. doi:10.1056/NEJM197904193001604.

124. Cover TL, Aber RC (1989) Yersinia enterocolitica. New England Journal of Medicine 321.

125. Bottone EJ (1997) Yersinia enterocolitica: the charisma continues. Clinical Microbiology Reviews 10: 257–276.

126. Biavati B, Sgorbati B, Scardovi Vittorio (1992) The Genus Bifidobacterium. In: Balows A, Trüpfer HG, Dworkin M, Harder W, Schleifer K-H, editors. The prokaryotes. New York and NY and Berlin and Heidelberg: Springer. pp. 816–833.

127. Saavedra J., Bauman N., Perman J., Yolken R., Oung I (1994) Feeding of Bifidobacterium bifidum and Streptococcus thermophilus to infants in hospital for prevention of diarrhoea and shedding of rotavirus. The Lancet 344: 1046–1049. doi:10.1016/S0140-6736(94)91708-6.

128. Fidel Jr. PL, Vazquez JA, Sobel JD (1999) Candida glabrata: Review of Epidemiology, Pathogenesis, and Clinical Disease with Comparison to C. albicans. Clinical Microbiology Reviews 12: 80–96.

129. Weems JJ (1992) Candida parapsilosis: Epidemiology, Pathogenicity, Clinical Manifestations, and Antimicrobial Susceptibility. Clinical Infectious Diseases 14: 756–766. doi:10.1093/clinids/14.3.756.

130. Pretorius IS (2000) Tailoring wine yeast for the new millennium: novel approaches to the ancient art of winemaking. Yeast 16: 675–729.

131. Eng RH., Drehmel R, Smith SM, Goldstein EJ. (1984) Saccharomyces cerevisiae infections in man. Medical Mycology 22: 403–407. doi:10.1080/00362178485380651.

132. McCusker JH, Clemons KV, Stevens DA, Davis R W (1994) Genetic characterization of pathogenic Saccharomyces cerevisiae isolates. Genetics 136: 1261–1269.

133. Munoz P, Bouza E, Cuenca-Estrella M, Eiros JM, Perez MJ, et al. (2005) Saccharomyces cerevisiae Fungemia: An Emerging Infectious Disease. Clinical Infectious Diseases 40: 1625–1634. doi:10.1086/429916.

134. Sobel JD, Vazquez J, Lynch M, Meriwether C, Zervos MJ (1993) Vaginitis due to Saccharomyces cerevisiae: epidemiology, clinical aspects, and therapy. Clinical Infectious Diseases 16: 93–99.

135. Collins MD, Jones D, Farrow JAE, Kilpper-Balz R, Schleifer KH (1984) Enterococcus avium nom. rev., comb. nov.; E. casseliflavus nom. rev., comb. nov.; E. durans nom. rev., comb. nov.; E. gallinarum comb. nov.; and E. malodoratus sp. nov. International Journal of Systematic Bacteriology 34: 220–223. doi:10.1099/00207713-34-2-220.

136. Patel R, Keating MR, Cockerill FR, Steckelberg JM (1993) Bacteremia Due to Enterococcus avium. Clinical Infectious Diseases 17: 1006–1011. doi:10.1093/clinids/17.6.1006.

137. Rosato A, Pierre J, Billot-Klein D, Buu-Hoi A, Gutmann L (1995) Inducible and constitutive expression of resistance to glycopeptides and vancomycin dependence in glycopeptide-resistant Enterococcus avium. Antimicrobial Agents and Chemotherapy 39: 830–833.

138. Svec P (2005) Enterococcus aquimarinus sp. nov., isolated from sea water. International Journal of Systematic and Evolutionary Microbiology 55: 2183–2187. doi:10.1099/ijs.0.63722-0.

139. Chen Y, Wu H, Lo H, Hsu W, Lin W, et al. (2012) Isolation, identification and characterization of lactic acid bacteria from Shao-jiou-luo (fermented zoned cerith), a traditional fermented food in Taiwan. Journal of Aquatic Food Product Technology: 121113080641001. doi:10.1080/10498850.2012.670191.

140. Castro A de, Montaño A, Casado F-J, Sánchez A-H, Rejano L (2002) Utilization of Enterococcus casseliflavus and Lactobacillus pentosus as starter cultures for Spanish-style green olive fermentation. Food Microbiology 19: 637–644. doi:10.1006/fmic.2002.0466.

141. Reid KC, Cockerill FR, Patel R (2001) Clinical and Epidemiological Features of Enterococcus casseliflavus/flavescens and Enterococcus gallinarum Bacteremia: A Report of 20 Cases. Clinical Infectious Diseases 32: 1540–1546. doi:10.1086/320542.

142. Altermann M, Rinklebe J, Merbach I, Körschens M, Langer U, et al. (2005) Chernozem—Soil of the Year 2005. Journal of Plant Nutrition and Soil Science 168: 725–740. doi:10.1002/jpln.200521814.

143. Jett B., Huycke MM, Gilmore M. (1994) Virulence of enterococci. Clinical Microbiology Reviews 7: 462–478.

144. Vankerckhoven V, van Autgaerden T, Vael C, Lammens C, Chapelle S, et al. (2004) Development of a Multiplex PCR for the Detection of asa1, gelE, cylA, esp, and hyl Genes in Enterococci and Survey for Virulence Determinants among European Hospital Isolates of Enterococcus faecium. Journal of Clinical Microbiology 42: 4473–4479. doi:10.1128/JCM.42.10.4473-4479.2004.

145. Gilad J, Borer A, Riesenberg K, Peled N, Shnaider A, et al. (1998) Enterococcus hirae septicemia in a patient with end-stage renal disease undergoing hemodialysis. European Journal of Clinical Microbiology & Infectious Diseases 17: 576–577. doi:10.1007/BF01708623.

146. Devriese LA, Chiers K, Herdt P de, Vanrompay D, Desmidt M, et al. (1995) Enterococcus hirae infections in psittacine birds: Epidemiological, pathological and bacteriological observations. Avian Pathology 24: 523–531. doi:10.1080/03079459508419091.

147. Tanasupawat S, Sukontasing S, Lee J-S (2008) Enterococcus thailandicus sp. nov., isolated from fermented sausage (’mum’) in Thailand. International Journal of Systematic and Evolutionary Microbiology 58: 1630–1634. doi:10.1099/ijs.0.65535-0.

148. Leroy F, Winter T de, Adriany T, Neysens P, Vuyst L de (2006) Sugars relevant for sourdough fermentation stimulate growth of and bacteriocin production by Lactobacillus amylovorus DCE 471. International Journal of Food Microbiology 112: 102–111. doi:10.1016/j.ijfoodmicro.2006.05.016.

149. Ogunbanwo S., Sanni A., Onilude A.A. (2003) Characterization of bacteriocin produced by Lactobacillus plantarum F1 and Lactobacillus brevis. African Journal of Biotechnology 2: 219–227.

150. Liu J, Schulz H, Brandl S, Miehtke H, Huwe B, et al. (2012) Short-term effect of biochar and compost on soil fertility and water status of a Dystric Cambisol in NE Germany under field conditions. Journal of Plant Nutrition and Soil Science 175: 698–707. doi:10.1002/jpln.201100172.

151. Benoit V, rie, Lebrihi A, Milli&#x000E8, re J-B, et al. (1997) Purification and Partial Amino Acid Sequence of Brevicin 27, a Bacteriocin Produced by Lactobacillus brevis SB27. Current Microbiology 34: 173–179. doi:10.1007/s002849900164.

152. Wada T, Noda M, Kashiwabara F, Jeon HJ, Shirakawa A, et al. (2009) Characterization of four plasmids harboured in a Lactobacillus brevis strain encoding a novel bacteriocin, brevicin 925A, and construction of a shuttle vector for lactic acid bacteria and Escherichia coli. Microbiology 155: 1726–1737. doi:10.1099/mic.0.022871-0.

153. Della Riccia DN, Bizzini F, Perilli MG, Polimeni A, Trinchieri V, et al. (2007) Anti-inflammatory effects of Lactobacillus brevis (CD2) on periodontal disease. Oral Diseases 13: 376–385. doi:10.1111/j.1601-0825.2006.01291.x.

154. Gionchetti P, Rizzello F, Venturi A, Brigidi P, Matteuzzi D, et al. (2000) Oral bacteriotherapy as maintenance treatment in patients with chronic pouchitis: A double-blind, placebo-controlled trial. Gastroenterology 119: 305–309. doi:10.1053/gast.2000.9370.

155. Guarneri T, Rossetti L, Giraffa G (2001) Rapid identification of Lactobacillus brevis using the polymerase chain reaction. Letters in Applied Microbiology 33: 377–381. doi:10.1046/j.1472-765X.2001.01014.x.

156. Kim J-W, Rajagopal S. (2001) Antibacterial Activities of Lactobacillus crispatus ATCC 33820 and Lactobacillus gasseri ATCC 33323. The Journal of Microbiology 39: 146–148.

157. Tahara T, Kanatani K (1997) Isolation and partial characterization of crispacin A, a cell-associated bacteriocin produced by Lactobacillus crispatus JCM 2009. FEMS Microbiology Letters 147: 287–290. doi:10.1111/j.1574-6968.1997.tb10255.x.

158. Boris S, Jimenez-Diaz R, Caso J., Barbes C (2001) Partial characterization of a bacteriocin produced by Lactobacillus delbrueckii subsp. lactis UO004, an intestinal isolate with probiotic potential. Journal of Applied Microbiology 91: 328–333. doi:10.1046/j.1365-2672.2001.01403.x.

159. Toba T, Yoshioka E, Itoh T (1991) Lacticin, a bacteriocin produced by Lactobacillus delbrueckii subsp. lactis. Letters in Applied Microbiology 12: 43–45. doi:10.1111/j.1472-765X.1991.tb00499.x.

160. Delley M, Mollet B, Hottinger H (1990) DNA Probe for Lactobacillus delbrueckii. Applied and Environmental Microbiology 56: 1967–1970.

161. Harty DW., Oakey H., Patrikakis M, Hume EB., Knox K. (1994) Pathogenic potential of lactobacilli. International Journal of Food Microbiology 24: 179–189. doi:10.1016/0168-1605(94)90117-1.

162. Pascual LM, Daniele MB, Giordano W, Pájaro MC, Barberis IL (2008) Purification and Partial Characterization of Novel Bacteriocin L23 Produced by Lactobacillus fermentum L23. Current Microbiology 56: 397–402. doi:10.1007/s00284-007-9094-4.

163. Ito Y, Kawai Y, Arakawa K, Honme Y, Sasaki T, et al. (2009) Conjugative Plasmid from Lactobacillus gasseri LA39 That Carries Genes for Production of and Immunity to the Circular Bacteriocin Gassericin A. Applied and Environmental Microbiology 75: 6340–6351. doi:10.1128/AEM.00195-09.

164. Kawai Y, Saito T, Kitazawa H, Itoh T (1998) Gassericin A; an Uncommon Cyclic Bacteriocin Produced by Lactobacillus gasseri LA39 Linked at N- and C-Terminal Ends. Bioscience, Biotechnology, and Biochemistry.

165. Sakamoto I, Igarashi M, Kimura K, Takagi A, Miwa T, et al. (2001) Suppressive effect of Lactobacillus gasseri OLL 2716 (LG21) on Helicobacter pylori infection in humans. Journal of Antimicrobial Chemotherapy 47: 709–710. doi:10.1093/jac/47.5.709.

166. Arakawa K, Kawai Y, Iioka H, Tanioka M, Nishimura J, et al. (2009) Effects of gassericins A and T, bacteriocins produced by Lactobacillus gasseri, with glycine on custard cream preservation. Journal of Dairy Science 92: 2365–2372. doi:10.3168/jds.2008-1240.

167. Casey P., Casey G., Gardiner G., Tangney M, Stanton C, et al. (2004) Isolation and characterization of anti-Salmonella lactic acid bacteria from the porcine gastrointestinal tract. Letters in Applied Microbiology 39: 431–438. doi:10.1111/j.1472-765X.2004.01603.x.

168. Delves-Broughton J, Blackburn P, Evans RJ, Hugenholtz J (1996) Applications of the bacteriocin, nisin. Antonie van Leeuwenhoek 69: 193–202. doi:10.1007/BF00399424.

169. Mattick AT., Hirsch A, Berridge N. (1947) FURTHER OBSERVATIONS ON AN INHIBITORY SUBSTANCE (NISIN) FROM LACTIC STREPTOCOCCI. The Lancet 250: 5–8. doi:10.1016/S0140-6736(47)90004-4.

170. Bolotin A, Wincker P, Mauger S, Jaillon O, Malarme K, et al. (2001) The Complete Genome Sequence of the Lactic Acid Bacterium Lactococcus lactis ssp. lactis IL1403. Genome Research 11: 731–753.

171. Fakhry S, Manzo N, D’Apuzzo E, Pietrini L, Sorrentini I, et al. (2009) Characterization of intestinal bacteria tightly bound to the human ileal epithelium. Research in Microbiology 160: 817–823. doi:10.1016/j.resmic.2009.09.009.

172. Lee JH, Valeriano VD, Shin Y-R, Chae JP, Kim G-B, et al. (2012) Genome Sequence of Lactobacillus mucosae LM1, Isolated from Piglet Feces. Journal of bacteriology 194: 4766. doi:10.1128/JB.01011-12.

173. Roos S, Karner F, Axelsson L, Jonsson H (2000) Lactobacillus mucosae sp. nov., a new species with in vitro mucus-binding activity isolated from pig intestine. International Journal of Systematic and Evolutionary Microbiology 50: 251–258. doi:10.1099/00207713-50-1-251.

174. Ahrné, Nobaek, Jeppsson, Adlerberth, Wold, et al. (1998) The normal Lactobacillus flora of healthy human rectal and oral mucosa. Journal of Applied Microbiology 85: 88–94. doi:10.1046/j.1365-2672.1998.00480.x.

175. Farrow JAE, Collins MD (1988) Notes: Lactobacillus oris sp. nov. from the Human Oral Cavity. International Journal of Systematic Bacteriology 38: 116–118. doi:10.1099/00207713-38-1-116.

176. Kõll P, Mändar R, Marcotte H, Leibur E, Mikelsaar M, et al. (2008) Characterization of oral lactobacilli as potential probiotics for oral health. Oral Microbiology and Immunology 23: 139–147. doi:10.1111/j.1399-302X.2007.00402.x.

177. Koll-Klais P, Mandar R, Leibur E, Marcotte H, Hammarstrom L, et al. (2005) Oral lactobacilli in chronic periodontitis and periodontal health: species composition and antimicrobial activity. Oral Microbiology and Immunology 20: 354–361. doi:10.1111/j.1399-302X.2005.00239.x.

178. Farrow JA., Phillips BA, Collins MD (1988) Nucleic acid studies on some heterofermentative lactobacilli: Description of Lactobacillus malefermentans sp.nov. and Lactobacillus parabuchneri sp.nov. FEMS Microbiology Letters 55: 163–168. doi:10.1111/j.1574-6968.1988.tb13927.x.

179. Coton M, Berthier F, Coton E (2008) Rapid identification of the three major species of dairy obligate heterofermenters Lactobacillus brevis , Lactobacillus fermentum and Lactobacillus parabuchneri by species-specific duplex PCR. FEMS Microbiology Letters 284: 150–157. doi:10.1111/j.1574-6968.2008.01206.x.

180. Axelsson LT, Chung TC, Dobrogosz WJ, Lindgren SE (1989) Production of a Broad Spectrum Antimicrobial Substance by Lactobacillus reuteri. Microbial Ecology in Health and Disease 2: 131–136. doi:10.3109/08910608909140210.

181. Gänzle MG, Holtzel A, Walter J, Jung G, Hammes WP (2000) Characterization of Reutericyclin Produced by Lactobacillus reuteri LTH2584. Applied and Environmental Microbiology 66: 4325–4333. doi:10.1128/AEM.66.10.4325-4333.2000.

182. Braat H, van den Brande J, van Tol E, Hommes Daan PM, van Deventer S (2004) Lactobacillus rhamnosus induces peripheral hyporesponsiveness in stimulated CD4+ T cells via modulation of dendritic cell function. The American Journal of Clinical Nutrition 80: 1618–1625.

183. Harty DWS, Patrikakis M, Knox KW (1993) Identification of Lactobacillus Strains Isolated from Patients with Infective Endocarditis and Comparison of their Surface-associated Properties with those of Other Strains of the Same Species. Microbial Ecology in Health and Disease 6: 191–201. doi:10.3109/08910609309141327.

184. Pascual LM, Daniele MB, Ruiz F, Giordano W, Pájaro C, et al. (2008) Lactobacillus rhamnosus L60, a potential probiotic isolated from the human vagina. The Journal of General and Applied Microbiology 54: 141–148. doi:10.2323/jgam.54.141.

185. Pessi T, Sutas Y, Hurme M, Isolauri E (2000) Interleukin-10 generation in atopic children following oral Lactobacillus rhamnosus GG. Clinical <html_ent glyph=\d q@amp;\d q ascii=\d q&\d q/> Experimental Allergy 30: 1804–1808. doi:10.1046/j.1365-2222.2000.00948.x.

186. Xiong T, Guan Q, Song S, Hao M, Xie M (2012) Dynamic changes of lactic acid bacteria flora during Chinese sauerkraut fermentation. Food Control 26: 178–181. doi:10.1016/j.foodcont.2012.01.027.

187. Peces R (1997) Relapsing bacteraemia due to Micrococcus luteus in a haemodialysis patient with a Perm-Cath catheter. Nephrology Dialysis Transplantation 12: 2428–2429. doi:10.1093/ndt/12.11.2428.

188. Bhunia A., Johnson MC, Ray B (1988) Purification, characterization and antimicrobial spectrum of a bacteriocin produced by Pediococcus acidilactici. Journal of Applied Bacteriology 65: 261–268. doi:10.1111/j.1365-2672.1988.tb01893.x.

189. Okereke A, Montville TJ (1991) Bacteriocin inhibition of Clostridium botulinum spores by lactic acid bacteria. Journal of Food Protection 54.

190. Piva A, Headon DR (1994) Pediocin A, a bacteriocin produced by Pediococcus pentosaceus FBB61. Microbiology 140: 697–702. doi:10.1099/00221287-140-4-697.

191. Barton LL, Rider ED, Coen RW (2001) Bacteremic Infection With Pediococcus: Vancomycin-Resistant Opportunist. PEDIATRICS 107: 775–776. doi:10.1542/peds.107.4.775.

192. Corcoran G., Gibbons N, Mulvihill T. (1991) Septicaemia caused by Pediococcus pentosaceus: A new opportunistic pathogen. Journal of Infection 23: 179–182. doi:10.1016/0163-4453(91)92190-G.

193. LOWY FD (1983) Staphylococcus epidermidis Infections. Annals of Internal Medicine 99: 834. doi:10.7326/0003-4819-99-6-834.

194. WADE JC (1982) Staphylococcus epidermidis: An Increasing Cause of Infection in Patients with Granulocytopenia. Annals of Internal Medicine 97: 503. doi:10.7326/0003-4819-97-4-503.

195. La Veach, Pfaller MA, Barrett M t., Koontz FP, Wenzel RP (1990) Vancomycin resistance in Staphylococcus haemolyticus causing colonization and bloodstream infection. Journal of Clinical Microbiology 28: 2064–2068.

196. Scheffer F, Meyer B (1963) Berührungspunkte der archaeologsichen und bodenkundlichen Forschung. Neue Ausgrabungen und Forschungen in Niedersachsen 1.

197. Shittu A (2004) Isolation and molecular characterization of multiresistant Staphylococcus sciuri and Staphylococcus haemolyticus associated with skin and soft-tissue infections. Journal of Medical Microbiology 53: 51–55. doi:10.1099/jmm.0.05294-0.

198. Kloos WE, Schleifer KH (1975) Isolation and Characterization of Staphylococci from Human Skin II. Descriptions of Four New Species: Staphylococcus warneri, Staphylococcus capitis, Staphylococcus hominis, and Staphylococcus simulans. International Journal of Systematic Bacteriology 25: 62–79. doi:10.1099/00207713-25-1-62.

199. Kloos WE, George CG, Olgiate JS, van Pelt L, McKinnon ML, et al. (1998) Staphylococcus hominis subsp. novobiosepticus subsp. nov., a novel trehalose- and N-acetyl-D-glucosamine-negative, novobiocin- and multiple-antibiotic-resistant subspecies isolated from human blood cultures. International Journal of Systematic Bacteriology 48: 799–812. doi:10.1099/00207713-48-3-799.

200. Shivaji S (2005) Bacillus arsenicus sp. nov., an arsenic-resistant bacterium isolated from a siderite concretion in West Bengal, India. International Journal of Systematic and Evolutionary Microbiology 55: 1123–1127. doi:10.1099/ijs.0.63476-0.

201. Batchelor DM (n.d.) AEROBIC SPORE-BEARING BACTERIA IN THE INTESTINAL TRACT OF CHILDREN.

202. Slepecky RA, Hemphill EH (1992) The Genus Bacillus - Nonmedical. In: Balows A, Trüpfer HG, Dworkin M, Harder W, Schleifer K-H, editors. The prokaryotes. New York and NY and Berlin and Heidelberg: Springer, Vol. 2. pp. 1663–1696.

203. Vary PS (1994) Prime time for Bacillus megaterium. Microbiology 140: 1001–1013. doi:10.1099/13500872-140-5-1001.

204. Heyrman J (2005) Study of mural painting isolates, leading to the transfer of “Bacillus maroccanus” and “Bacillus carotarum” to Bacillus simplex, emended description of Bacillus simplex, re-examination of the strains previously attributed to “Bacillus macroides” and description of Bacillus muralis sp. nov. International Journal of Systematic and Evolutionary Microbiology 55: 119–131. doi:10.1099/ijs.0.63221-0.

205. Hu X, van der Auwera G, Timmery S, Zhu L, Mahillon J (2009) Distribution, Diversity, and Potential Mobility of Extrachromosomal Elements Related to the Bacillus anthracis pXO1 and pXO2 Virulence Plasmids. Applied and Environmental Microbiology 75: 3016–3028. doi:10.1128/AEM.02709-08.

206. Bargabus R., Zidack N., Sherwood J., Jacobsen B. (2002) Characterisation of systemic resistance in sugar beet elicited by a non-pathogenic, phyllosphere-colonizing Bacillus mycoides, biological control agent. Physiological and Molecular Plant Pathology 61: 289–298. doi:10.1006/pmpp.2003.0443.

207. Landy M, Warren GH, RosenmanM SB, Colio LG (1948) Bacillomycin: An Antibiotic from Bacillus subtilis Active against Pathogenic Fungi. Experimental Biology and Medicine 67: 539–541. doi:10.3181/00379727-67-16367.

208. Pusey PL (1984) Postharvest Biological Control of Stone Fruit Brown Rot by Bacillus subtilis. Plant Disease 68: 753. doi:10.1094/PD-69-753.

209. Schnepf E, Crickmore N, van Rie J, Lereclus D, Baum j., et al. (1998) Bacillus thuringiensis and Its Pesticidal Crystal Proteins. Microbiology and Molecular Biology Reviews 62: 775–806.

210. LECHNER S, MAYR R, FRANCIS KP, PRUss BM, KAPLAN T, et al. (1998) Bacillus weihenstephanensis sp. nov. is a new psychrotolerant species of the Bacillus cereus group. International Journal of Systematic Bacteriology 48: 1373–1382. doi:10.1099/00207713-48-4-1373.

211. Prü\s s BM, Dietrich R, Nibler B, Märtelbauer E, Scherer S (1999) The Hemolytic Enterotoxin HBL Is Broadly Distributed among Species of the Bacillus cereusGroup. Applied and Environmental Microbiology 65: 5436–5442.

212. Stenfors LP, Mayr R, Scherer S, Granum PE (2002) Pathogenic potential of fifty Bacillus weihenstephanensis strains. FEMS Microbiology Letters 215: 47–51. doi:10.1111/j.1574-6968.2002.tb11368.x.

213. Barash JR, Tang TWH, Arnon SS (2005) First Case of Infant Botulism Caused by Clostridium baratii Type F in California. Journal of Clinical Microbiology 43: 4280–4282. doi:10.1128/JCM.43.8.4280-4282.2005.

214. Harvey SM, Sturgeon J, Dassey DE (2002) Botulism Due to Clostridium baratii Type F Toxin. Journal of Clinical Microbiology 40: 2260–2262. doi:10.1128/JCM.40.6.2260-2262.2002.

215. Montecucco C, Rossetto O, Popoff MR (2006) Neurotoxigenic Clostridia. In: Dworkin M, Falkow S, Rosenberg E, Schleifer K-H, Stackebrandt E, editors. The Prokaryotes. New York and NY: Springer US. pp. 679–697.

216. Hippe H, Andreesen JR, Gottschalk G (1992) The Genus Clostridium-Nonmedical. In: Balows A, Trüpfer HG, Dworkin M, Harder W, Schleifer K-H, editors. The prokaryotes. New York and NY and Berlin and Heidelberg: Springer. pp. 1800–1866.

217. Nishida S, Tamai K, Yamagishi T (1964) TAXONOMY OF CLOSTRIDIUM BIFERMENTANS AND CLOSTRIDIUM SORDELLII I. Their Toxigenicity, Urease Activity, and Sporulating Potency. Journal of bacteriology 88: 1641–1646.

218. Aureli P, Fenicia L, Pasolini B, Gianfranceschi M, McCroskey LM, et al. (1986) Two Cases of Type E Infant Botulism Caused by Neurotoxigenic Clostridium butyricum in Italy. Journal of Infectious Diseases 154: 207–211. doi:10.1093/infdis/154.2.207.

219. Finegold SM, Downes J, Summanen PH (2012) Microbiology of regressive autism. Anaerobe 18: 260–262. doi:10.1016/j.anaerobe.2011.12.018.

220. Finegold SM, Song Y, Liu C, Hecht DW, Summanen P, et al. (2005) Clostridium clostridioforme: a mixture of three clinically important species. European Journal of Clinical Microbiology & Infectious Diseases 24: 319–324. doi:10.1007/s10096-005-1334-6.

221. Song Y, Liu C, Molitoris DR, Tomzynski TJ, Lawson PA, et al. (2003) Clostridium bolteae sp. nov., Isolated from Human Sources. Systematic and Applied Microbiology 26: 84–89. doi:10.1078/072320203322337353.

222. PALOP MLL, VALLES S, PINAGA F, FLORS A (1989) Isolation and Characterization of an Anaerobic, Cellulolytic Bacterium, Clostridium celerecrescens sp. nov. International Journal of Systematic Bacteriology 39: 68–71. doi:10.1099/00207713-39-1-68.

223. Glazunova OO, Raoult D, Roux V (2005) First Identification of Clostridium celerecrescens in Liquid Drained from an Abscess. Journal of Clinical Microbiology 43: 3007–3008. doi:10.1128/JCM.43.6.3007-3008.2005.

224. Pan-Hou HS., Kajikawa Y, Imura N (1982) Characterization of organomercury-decomposing activity in cell extract of mercury-resistant Clostridium cochlearium T-2P. Ecotoxicology and Environmental Safety 6: 82–88. doi:10.1016/0147-6513(82)90082-3.

225. Reitzer R, Gruber K, Jogl G, Wagner UG, Bothe H, et al. (1999) Glutamate mutase from Clostridium cochlearium: the structure of a coenzyme B12-dependent enzyme provides new mechanistic insights. Structure 7: 891–902. doi:10.1016/S0969-2126(99)80116-6.

226. Greetham HL (2003) Clostridium colicanis sp. nov., from canine faeces. International Journal of Systematic and Evolutionary Microbiology 53: 259–262. doi:10.1099/ijs.0.02260-0.

227. Horn N (1987) Clostridium disporicum sp. nov., a Saccharolytic Species Able to Form Two Spores per Cell, Isolated from a Rat Cecum. International Journal of Systematic Bacteriology3 37: 398–401.

228. Plassart C, Mauvais F, Heurté J, Sautereau J, Legeay C, et al. (2013) First case of intra-abdominal infection with Clostridium disporicum. Anaerobe 19: 77–78. doi:10.1016/j.anaerobe.2012.12.002.

229. Evvyernie D, Yamazaki S, Morimoto K, Karita S, Kimura T, et al. (2000) Identification and characterization of Clostridium paraputrificum M-21, a chitinolytic, mesophilic and hydrogen-producing bacterium. Journal of Bioscience and Bioengineering 89: 596–601. doi:10.1016/S1389-1723(00)80063-8.

230. Dernby KG, Blanc J (1921) On the growth and the proteolytic enzymes of certain anaerobes. Journal of bacteriology 6: 419–430.

231. Smith LD. (1992) The Genus Clostridium-Medical. In: Balows A, Trüpfer HG, Dworkin M, Harder W, Schleifer K-H, editors. The prokaryotes. New York and NY and Berlin and Heidelberg: Springer. pp. 1867–1878.

232. Songer GJ (2010) Clostridia as agents of zoonotic disease. Veterinary Microbiology.

233. Miech RP (2005) Pathophysiology of Mifepristone-Induced Septic Shock Due to Clostridium sordellii. Annals of Pharmacotherapy 39: 1483–1488. doi:10.1345/aph.1G189.

234. Aldape MJ, Bryant AE, Stevens DL (2006) Clostridium sordellii Infection: Epidemiology, Clinical Findings, and Current Perspectives on Diagnosis and Treatment. Clinical Infectious Diseases 43: 1436–1446. doi:10.1086/508866.

235. Inkster T, Cordina C, Siegmeth A (2011) Septic arthritis following anterior cruciate ligament reconstruction secondary to Clostridium sporogenes; a rare clinical pathogen. Journal of Clinical Pathology 64: 820–821. doi:10.1136/jcp.2010.084434.

236. MacLennan JD (1962) THE HISTOTOXIC CLOSTRIDIAL INFECTIONS OF MAN. Bacteriology Reviews 26: 177–274.

237. Priest FG, Goodfellow M, Todd C (1988) A Numerical Classification of the Genus Bacillus. Microbiology 134: 1847–1882. doi:10.1099/00221287-134-7-1847.

238. Hakizimana JD, Gryzenhout M, Coutinho TA, van den Berg N (2011) Endophytic diversity in Persea americana (avocado) trees and their ability to display biocontrol activity against Phytophthora cinnamomi. Proc. VII World Avocado Congress. pp. 1–10.

239. Pradhan AK, Pradhan N, Sukla LB, Panda PK, Mishra BK (2013) Inhibition of pathogenic bacterial biofilm by biosurfactant produced by Lysinibacillus fusiformis S9. Bioprocess and Biosystems Engineering. doi:10.1007/s00449-013-0976-5.

240. Adelowo OO, Fagade OE (2012) Phylogenetic characterization, antimicrobial susceptibilities, and mechanisms of resistance in bacteria isolates from a poultry waste-polluted river, southwestern Nigeria. TURKISH JOURNAL OF BIOLOGY 36: 37–45.

241. Hall-Stoodley L, Costerton JW, Stoodley P (2004) Bacterial biofilms: from the Natural environment to infectious diseases. Nature Reviews Microbiology 2: 95–108. doi:10.1038/nrmicro821.

242. ALEXANDER B, Priest FG (1989) Bacillus glucanolyticus, a New Species That Degrades a Variety of -Glucans. International Journal of Systematic Bacteriology 39: 112–115. doi:10.1099/00207713-39-2-112.

243. Pridal A (2002) Effects of three bacterial species on Bombus terrestris larvae under laboratory conditions. Acta Universitatis Agriculturae et Silviculturae Mendelianae Brunensis 50.

244. Ferrand J, Hadou T, Selton-Suty C, Goehringer F, Sadoul N, et al. (2013) Cardiac Device-Related Endocarditis Caused by Paenibacillus glucanolyticus. Journal of Clinical Microbiology 51: 3439–3442. doi:10.1128/JCM.00864-13.

245. Scheldeman P, Goossens K, Rodriguez-Diaz M, Pil A, Goris J, et al. (2004) Paenibacillus lactis sp. nov., isolated from raw and heat-treated milk. International Journal of Systematic and Evolutionary Microbiology 54: 885–891.

246. HEYNDRICKX M, VANDEMEULEBROECKE K, Scheldeman P, KERSTERS K, VOS P de, et al. (1996) A Polyphasic Reassessment of the Genus Paenibacillus, Reclassification of Bacillus lautus (Nakamura 1984) as Paenibacillus lautus comb. nov. and of Bacillus peoriae (Montefusco et al. 1993) as Paenibacillus peoriae comb. nov., and Emended Descriptions of P. lautus and of P. peoriae. International Journal of Systematic Bacteriology 46: 988–1003. doi:10.1099/00207713-46-4-988.
